# Maximizing impact: the power of early HPV vaccination for long-term protection―lessons from a systematic review and meta-regression analysis

**DOI:** 10.1093/pubmed/fdag002

**Published:** 2026-01-30

**Authors:** Adoración Navarro-Torné, Emmanuel Aris, Andrea Callegaro, Bernd Benninghoff, Huifeng Yun, Volker Vetter

**Affiliations:** Viral Non-Respiratory, Vaccines and Infectious Disease Epidemiology, GSK, Tres Cantos 28760, Spain; Real-World Biostatistics, GSK, Wavre 1300, Belgium; Statistics, GSK, Rixensart 1330, Belgium; A&N Immugen, Egloffstein 91349, Germany; Viral Non-Respiratory, Vaccines and Infectious Disease Epidemiology, GSK, Rockville, MD 20850, USA; Global Medical Lead, GSK, Wavre 1300, Belgium

**Keywords:** vaccination, systematic review, public health

## Abstract

**Background:**

This systematic review and meta-regression analysis assessed the impact of human papillomavirus 16/18 (HPV16/18)-AS04 vaccine (Cervarix^®^) on advanced cervical lesions, including grade 3 cervical intraepithelial neoplasia or worse (CIN3, CIN3+), or cervical cancer, highlighting age-at-vaccination–dependent vaccine efficacy and effectiveness.

**Methods:**

Studies reporting HPV16/18-AS04 vaccine efficacy or effectiveness were included with an intervention group receiving HPV16/18-AS04 vaccine and comparator group receiving placebo, another vaccine or being unvaccinated. Of 53 articles identified, nine were selected. Meta-analysis and meta-regression models with random effects and data-driven model selection determined vaccine effects (VEs) and impactful covariates.

**Results:**

HPV16/18-AS04 vaccine effectively prevented advanced cervical premalignant lesions and cancer in adolescent girls and women vaccinated at 12–25 years. Combined randomized controlled trials and observational studies VEs on CIN3+ ranged between 76.78% (95% CI 28.15–92.49) for HPV16/18 and 56.19% (95% CI 24.76–74.49) irrespective of HPV type. Vaccine effectiveness was greatest in those vaccinated at the youngest ages.

**Conclusions:**

HPV16/18-AS04 vaccine provides long-term protection against cervical premalignant lesions and cervical cancer in both controlled and real-world settings, particularly when administered at younger ages. The evidence urges policymakers and the community to ensure HPV vaccination begins at the youngest recommended ages.

## Introduction

Human papillomavirus (HPV) is the most common sexually transmitted virus, causing infections worldwide, with the highest prevalence among women under 25 years of age, corresponding to the onset of exposure following sexual debut.^1^ HPV infections usually clear within 12–24 months post-infection.^2^ Long-term persistent infection with high-risk (HR) HPV types (mainly 16, 18, 31, 33, 35, 39, 45, 51, 52, 56, 58, 59, and 68) increases the risk of oncogenic progression, potentially leading to invasive cancer.^3–5^ HPV 16 and 18 account for 77% of cervical cancers worldwide, with types 31, 33, 45, 52, and 58 causing another 18% of cases.^6^

Cervical cancer is the fourth most common cancer among women worldwide, with an estimated > 600 000 new cases ever year and > 340 000 deaths in 2020.^7^ Other anogenital HPV-related cancers include vulvar, vaginal, penile, and anal cancers. Additionally, HPV-positive head and neck cancers, particularly oropharyngeal tumors, have increased significantly in recent decades.^8^

HPV vaccines, first approved in 2006, have been gradually introduced into national immunization programmes (NIPs) worldwide. As sexual activity constitutes the current paradigm for HR HPV acquisition, prophylactic vaccination is recommended before sexual debut, from as early as 9 years of age in some countries,^9^ and, in many, with a gender-neutral approach.^10^ However, the vaccination coverage among adolescents remains suboptimal and often delayed, failing to meet established targets.^11^^,^^12^

The World Health Organization (WHO) has established a global health strategy to eliminate cervical cancer as a public health problem, aiming for 90% of girls to be fully vaccinated with an HPV vaccine by the age of 15 years; 70% of women screened by ages 35 and again by 45 years; and 90% of women with pre-cancer treated and those with invasive cancer managed by 2030.^13^

The HPV16/18-AS04 vaccine (Cervarix^®^) is a bivalent adjuvanted vaccine manufactured by GSK, indicated for the prevention of premalignant anogenital lesions (cervical, vulvar, vaginal, and anal) and cervical and anal cancers caused by HPV types 16 and 18.^14^ To compile evidence on the long-term effects of HPV16/18-AS04 on grade 3 cervical intraepithelial neoplasia or worse (CIN3, CIN3+) and cervical cancer, a systematic literature review and meta-analysis/meta-regression analyses were conducted.

## Methods

### Search strategy and selection criteria

The systematic review aimed to collect data from long-term follow-up studies of randomized controlled trials (RCTs), long-term observational studies and national surveillance data from countries that implemented HPV16/18-AS04 in their NIPs. Additionally, it aimed to determine a summary estimate of HPV16/18-AS04 efficacy/effectiveness against CIN3+ and assess variables impacting the outcome through meta-regression.

Included studies met these criteria: (i) reported HPV16/18-AS04 efficacy (RCTs) or effectiveness (observational studies: cohort, cross-sectional, case–control, longitudinal, population-based surveillance) against cervical cancer and/or CIN3/CIN3+; (ii) comparator group received a placebo, another vaccine or no vaccination; (iii) intervention group received ≥1 dose of HPV16/18-AS04; and (iv) peer-reviewed articles with abstracts in English, French, Spanish, Portuguese, German or Italian. Grey literature was excluded. No geographical or race limits were applied.

Journal articles published between 1 January 2000 to 21 June 2022 in PubMed, EMBASE, Scopus and Cochrane CENTRAL were considered. The search focused on ‘bivalent human papillomavirus vaccine’, ‘vaccine efficacy,’ ‘vaccine effectiveness,’ ‘population surveillance’ and ‘cervical intraepithelial neoplasia Grade 3’ (Supplementary Table S1). The study was registered in EU PAS (ENCePP), #EUPAS1000000026.

### Data analysis

A reviewer searched the databases and exported references to Endnote^®^, wherein duplicates were removed. Titles and abstracts were screened by one reviewer (ANT), whereas full-text reports were screened by two independent reviewers (ANT, BB), noting reasons for exclusion. Disagreements were resolved by a third reviewer (NK). Inter-reviewer agreement was determined using Cohen’s kappa coefficient.^15^ When several papers referred to the same population (even partially) and scope, the most recent and comprehensive paper was selected.

Two independent reviewers (ANT, MM) extracted the data, which were cross-checked and confirmed. Discrepancies were resolved by consensus, with no final disagreements. Data extraction focused on vaccine efficacy (RCTs) or effectiveness (observational studies) against CIN3+ as the primary outcome. When vaccine effectiveness was not reported, odds ratios (ORs) and incidence rate ratios (IRRs) were used, with effectiveness calculated as (1 − OR)*100 or (1 − IRR)*100, respectively. For this study, vaccine effects (VEs) refer to a combination of vaccine efficacy and effectiveness. The age at first vaccination or age range at the time of vaccination for each subgroup has been detailed in the legend/footnote of each figure. This manuscript adheres to the 2020 PRISMA checklist for systematic reviews.^16^ Publication bias assessment was not pursued due to fewer than the recommended 10 studies in the meta-analysis.^17^

For quantitative analysis, combined effects of VE on CIN3+ from RCTs and observational studies were considered, with sensitivity analyses conducted to assess variations based on study design. Regarding the analytical cohort, data were reported for the Total Vaccinated Cohort (TVC; regardless of baseline HPV status and receiving ≥1 dose) and the TVC-naïve (HPV-naïve at first vaccination) due to differences in vaccine efficacy/effectiveness. In meta-regression analyses, these cohorts were considered separately (binary covariate). Groups vaccinated with ‘three doses’ and ‘≥1 dose’ were pooled if ≥75% of the ‘≥1 dose’ group had received three doses. Other variables included ‘age at first vaccination’, ‘time since vaccination’ and ‘HPV type.’ Outcomes were stratified by ‘vaccine types’ (HPV 16/18) and ‘irrespective of HPV type’. Studies reporting vaccine effectiveness based on histological diagnosis alone^18^ or against a composite index of 14 HR HPV types were classified under ‘irrespective of HPV type.’ ‘Study correlation’ was introduced as a dummy variable to adjust for potential correlation in studies.

All statistical analyses were conducted using R software version 4.1.3 (metafor_3.8–1 and metadata_1.2–0 packages) with random-effects models. The strategy included (i) meta-analysis was fitted; (ii) univariate meta-regression analyses were then fitted to assess each covariate independently; and (iii) multiparametric meta-regression analysis was conducted to adjust for selected covariates. The Akaike Information Criterion (AIC) was used to compare different multiparametric models for the best prediction.

The *I^2^* statistic was used to quantify heterogeneity. Random effects were shared within subgroups from the same study. Analyses were pre-specified and organized to address different questions using different datasets. The main text results correspond to the questions ‘What is the combined overall efficacy/effectiveness of HPV16/18-AS04 against CIN3+ caused by vaccine HPV types?’ (RCTs and observational studies combined) (analysis 1) and ‘What is the combined overall efficacy/effectiveness of HPV16/18-AS04 against CIN3+ irrespective of HPV type?’ (RCTs and observational studies combined) (analysis 2). Results for other subgroups (analyses 3–6) are in Supplementary material.

The risk of bias was assessed using the Cochrane risk of bias tool for RCTs (RoB 2)^19^ and the Cochrane ROBINS-I tool for observational epidemiological studies.^20^ For papers reporting both RCTs and observational studies, the appropriate tool was used to gauge the quality of each component.

## Results

The database search identified 2803 potential eligible articles, including one from a hand search (Supplementary Fig. S1). After removing 913 duplicates, 1890 articles remained. Based on the eligibility, inclusion or exclusion criteria, 1837 articles were excluded, leaving 53 for full-text review. Nine of these met the inclusion criteria: five follow-up studies of RCTs,^21–25^ three retrospective population-based registry-linked studies^18^^,^^26^^,^^27^ and one observational post-hoc long-term follow-up study of an RCT^28^ (Table 1; Supplementary Table S2). Two RCTs also had an observational component.^24^^,^^25^ The studies were conducted in Japan,^23^ Costa Rica,^24^^,^^25^ Finland,^28^ Scotland,^18^ England^24^^,^^25^ and multiple countries.^21^^,^^22^ Seven studies were selected for quantitative synthesis,^18^^,^^22–25^^,^^27^^,^^28^ whereas two were selected for narrative review alone^21^^,^^26^ (Supplementary Table S3; Supplementary material).

**Table 1:** Summary of characteristics of selected studies.

Author, Year	Country	Study period	Study design	Study population	Age at first vaccination (years)	Vaccine coverage	Number of doses	Type of outcomes	Case-counting start
Wheeler, 2012^19^	Multi-country (US, Australia, Belgium, Brazil, Canada, Finland, Germany, Italy, Philippines, Spain, Taiwan, Thailand, UK)	June 2004–June 2008	RCT (4-year follow-up)	Females with ≤ 6 lifetime sexual partners (except in Finland), regardless of baseline HPV DNA status, HPV16 or HPV18 serostatus or cytology. *N* = 16 114, 11 644 and 18 644 women were included in the ATP-E (vaccine arm *n* = 8067, control arm *n* = 8047), TVC-naïve (vaccine arm *n* = 5824, control arm *n* = 5820), and TVC cohorts (vaccine arm *n* = 9319, control arm *n* = 9325), respectively. 16% of participants (3034 of 18 644) were lost to follow-up by the end of the study	15–25	NA	Participants considered for the analysis, three doses-ATP-E cohort At least one dose: TVC-naïve and TVC	Vaccine efficacy	Day after first vaccination for TVC-naïve and TVC and the day after third vaccination for ATP-E cohort
Lehtinen, 2012^20^	Multi-country (US, Australia, Belgium, Brazil, Canada, Finland, Germany, Italy, Philippines, Spain, Taiwan, Thailand, UK)	June 2004–June 2008	RCT (4-year follow-up)	Females with ≤ 6 lifetime sexual partners (except in Finland), regardless of baseline HPV DNA status, HPV16 or HPV18 serostatus or cytology. Completed study: TVC: HPV arm *n* = 7798, control arm *n* = 7811 TVC-naïve: HPV arm *n* = 1879, control arm *n* = 2315 ATP-E: HPV arm *n* = 6815, control arm *n* = 6769	15–25	NA	Participants considered for the analysis, three doses-ATP-E cohort At least one dose: TVC-naïve and TVC	Vaccine efficacy	Day after first vaccination for TVC-naïve and TVC and the day after third vaccination for ATP-E cohort
Konno, 2014^21^	Japan	October 2009–April 2013	RCT (4-year follow-up)	Healthy females not screened before enrolment for baseline serological, cytological or HPV DNA status. TVC-combined: HPV arm *n* = 519, control arm *n* = 521 ATP cohort for efficacy-combined: HPV arm *n* = 499, control arm *n* = 498 TVC-naïve–combined: HPV arm *n* = 281, control arm *n* = 284	20–25	NA	Participants considered for the analysis if at least one dose: TVC-naïve and TVC	Vaccine efficacy	Day after receipt of the first vaccine dose for the TVC-naïve and TVC (up to 4 years follow-up) and the day after third vaccination for ATP-E cohort

(*Continued*)

**Table 1:** Continued

Author, Year	Country	Study period	Study design	Study population	Age at first vaccination (years)	Vaccine coverage	Number of doses	Type of outcomes	Case-counting start
Lehtinen, 2017^26^	Finland	Enrolment: June 2003/2005 and May 2004–April 2005. Follow-up: 2009–2015	Cohort study	18–19-year-old unvaccinated women *n* = 15 627 16–17-year-old vaccinated women *n* = 2401 PATRICIA trial 16–17-year-old vaccinated women *N* = 64 HPV-012 trial	15–25 PATRICIA trial 10–25 HPV-012 trial	NA	Participants considered for analysis if at least one dose administered (TVC)	Vaccine effectiveness	Day after first vaccination (up to 10 years post-vaccination follow-up)
Porras, 2020^22^	Costa Rica	June 2004–December 2005 (RCT); follow-up: March 2009–July 2012 (total 11 years)	RCT (up to year 4) and cohort study (no randomization) (up to year 11)	Healthy women (HPV16/18 DNA-negative at months 0 and 6, who did not undergo biopsy or LEEP during the vaccination phase) HPV vaccine group *n* = 2635, control group (0–4 years RCT) *n* = 2677 HPV vaccine group *n* = 2073, unvaccinated group in cohort analysis (7–11 years) *n* = 2530	18–25	NA	Three doses	Vaccine efficacy Vaccine effectiveness	Day after first vaccination (up to year 11 of follow-up)
Shing, 2022^23^	Costa Rica	June 2004–December 2005 (RCT); follow-up: March 2009–July 2012 (total 11 years)	RCT (up to year 4) and cohort study (no randomization) (up to year 11)	*n* = 3491 in HPV vaccine group and *n* = 3512 in control arm (CIN3+ endpoint, years 1–4 of follow-up) *n* = 2826 in HPV vaccine group and *n* = 2592 in unvaccinated control arm (CIN3+ endpoint, years 7–11 of follow-up) Note: Analyses included all participants with at least one follow-up visit in the respective period and excluded participants with a previous endpoint (CIN2+, CIN3+) (i.e. mITT)	18–25	NA	At least one dose (mITT)	Vaccine efficacy Vaccine effectiveness	Day after first vaccination (up to year 11 of follow-up)
Palmer, 2019^16^	Scotland (UK)	Between 1 January 1988 and 5 June 1996 for screening; extraction date: August 2017	Retrospective population-based study	Routinely vaccinated girls aged 12–13 years (born between 1 January 1988 and 5 June 1996); catch-up campaign for vaccinated women (born during 1991–94, age 14–17 at vaccination); unvaccinated women (born during 1988–90, age 18–20 in 2008) screened at age 20; *N* = 138 692 women screened at age 20	12–13 14 15 16 17 ≥18	90% at age 13 (1995 birth cohort)	Three, two or one dose	OR	NA

(*Continued*)

**Table 1:** Continued

Author, Year	Country	Study period	Study design	Study population	Age at first vaccination (years)	Vaccine coverage	Number of doses	Type of outcomes	Case-counting start
Falcaro, 2021^24^	England (UK)	January 2006–June 2019; data extracted on 26 January 2021	Retrospective population-based database study	Vaccine-eligible women (seven birth cohorts); unvaccinated cohort (born between 1 May 1989 and 31 August 1990); 13.7 million-years of follow-up of women aged 20 years to < 30 years in the three vaccinated cohorts	12–13 14–16 16–18	Routine cohort: 85.9%–90.6% for 2008–2009 and 2011–2012 Catch-up cohort: 55.6%–81.9% one dose: 60.5%–88.7% three doses: 44.8%–84.9%	At least one dose, three doses	Adjusted IRR	NA
Rebolj, 2022^25^	England (UK)	2013–2018	Retrospective population-based database study	Women eligible for catch-up vaccination (14–17 years) and received HR-HPV test at 25 years *N* = 64 274 overall results of women tested; *N* = 42 384 genotyped results	Vaccinated cohort 24–25; Unvaccinated cohort 26–29	40%–75% depending on the birth cohort	Data on individual vaccination status unavailable	Vaccine effectiveness	NA

ATP-E = according-to-protocol for efficacy cohort; CIN2+ = grade 2 cervical intraepithelial neoplasia or worse; CIN3+ = grade 3 cervical intraepithelial neoplasia or worse; DNA = deoxyribonucleic acid; HPV = human papillomavirus; HR-HPV = high-risk human papillomavirus; IRR = incident relative risk (or risk ratio); LEEP = loop electrosurgical excision procedure; mITT = modified intention to treat; N = total (overall); n = number of participants in each arm; NA = not applicable; OR = odds ratio; RCT = randomized controlled trial; TVC = total vaccinated cohort; UK=United Kingdom; US=United States; VE = vaccine effects.

For this study, vaccine efficacy refers to outcomes/results recorded in controlled conditions (RCTs), vaccine effectiveness refers to outcomes/results recorded in real-world conditions (observational studies) and VE is a combination of vaccine efficacy and vaccine effectiveness.

Wheeler (2012) and Falcaro (2021) were excluded from the quantitative analysis as they did not align with the focus of main analysis, which assessed the efficacy/effectiveness of HPV16/18-AS04 vaccine on CIN3+ caused by vaccine types (HPV16 and/or 18) (analysis 1) and irrespective of HPV type (analysis 2). Wheeler (2012) evaluated CIN3+ associated with non-vaccine HPV types, while Falcaro (2021) addressed CIN3 and cervical cancer outcomes.

The inter-rater reliability calculation yielded a Cohen’s kappa coefficient of 82.3% (95% CI 63.1–100), indicating near-perfect agreement.^15^ No studies were excluded based on quality. Post-hoc studies of RCTs by Lehtinen (2012),^22^ Porras (2020),^24^ and Shing (2022)^25^ showed low bias risk, with double-blinding maintained beyond the 3-year RCT duration up to 48 months, except for Konno (2014)^23^ which was unblinded at 36 months. Follow-up completeness was high, and losses were not selective, with balanced arms at study completion. Observational studies had at least a moderate risk of bias, with two of the five having a serious risk of bias^18^^,^^27^ due to high-risk domains (mainly confounding and outcome information; Supplementary Tables S4–S6, Figs. S2–S3). A meta-analysis was conducted to determine the combined efficacy/effectiveness of HPV16/18-AS04 against CIN3+ caused by vaccine HPV types (HPV16/18), pooling vaccine efficacy data from two RCT follow-up studies, Lehtinen (2012)^22^ and Porras (2020),^24^ and vaccine effectiveness data from two observational studies, Shing (2022)^25^ and Rebolj (2022).^27^ The observational component of Shing (2022)^23^ captured the long-term follow-up of the Costa Rica Vaccine Trial, although participants partially overlapped with those of Porras (2020),^22^ using different approach to the analytical cohorts (TVC *vs* TVC-naïve). Random effects were used to account for the partial overlap, and a ‘study correlation’ variable was introduced.

Remarkably, the combined overall VE from RCTs and observational studies on CIN3+ caused by HPV16/18 was 76.78% (95% CI 28.15–92.49) (Fig. S4), whereas it was 56.19% (95% CI 24.76–74.49%) when CIN3+ was caused by any HPV type (Table 2).

**Table 2:** Pooled long-term vaccine effects and impactful covariates.

Analysis	Outcome	Meta-analysis Vaccine effect (95% CI)	Meta-regression analysis Impactful covariates
**HPV16/18**			
Analysis 1 (RCTs, observational studies)	Combined vaccine effects	76.78% (28.15–92.49)	Age at first vaccination, analytical cohort^a^
Analysis 3 (RCTs)	Vaccine efficacy	47.84% (24.51–63.96)	Age at first vaccination, analytical cohort^a^
Analysis 4 (Observational studies)	Vaccine effectiveness	78.35% (―123.19–97.90)	NA^b^
**Irrespective of HPV type**			
Analysis 2 (RCTs, observational studies)	Combined vaccine effects	56.19% (24.76–74.49)	Age at first vaccination, analytical cohort^a^
Analysis 5 (RCTs)	Vaccine efficacy	48.89% (19.84–67.41)	Age at first vaccination, analytical cohort^a^
Analysis 6 (Observational studies)	Vaccine effectiveness	65.45% (42.02–79.41)	Age at first vaccination, time since vaccination

CI = confidence interval; HPV = human papillomavirus; NA = not applicable; RCT = randomized controlled trial; TVC = total vaccinated cohort; VE = vaccine effects;

For this study, vaccine efficacy refers to outcomes/results recorded in controlled conditions (RCTs), vaccine effectiveness refers to outcomes/results recorded in real-world conditions (observational studies) and VE is a combination of vaccine efficacy and vaccine effectiveness.

^a^Analytical cohorts: TVC (irrespective of baseline HPV status) and TVC-naïve (HPV-naïve at baseline) cohorts. ^b^The data-driven multiparametric meta-regression selection did not identify any stable model.

The univariate meta-regression analyses of the first scenario (analysis 1) showed a strong association of VE with ‘age at first vaccination’ (*P* = .01), ‘study design’ (RCT follow-up *vs* observational study, *P* = .001) and ‘time since vaccination’ (0–4 years *vs* 7–11 years of follow-up, *P* = .001) (Supplementary Figs. S5―S8). VE decreased with age at first vaccination, was lower in RCTs compared with observational studies and was larger within ‘0–4’ years (RCTs) post-vaccination compared with ‘7–11’ years (observational studies). VE was also larger in TVC-naïve than in TVC.

**Figure 1:**
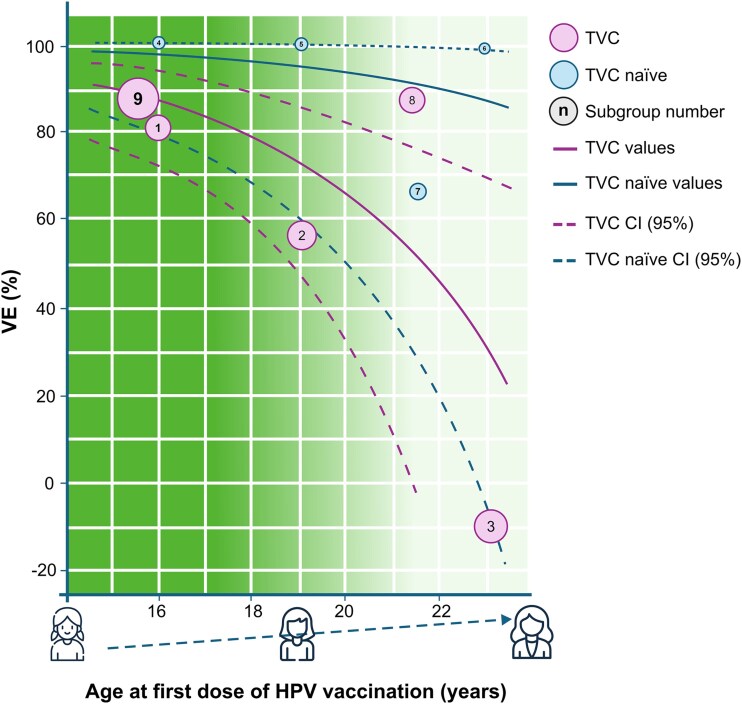
Predicted VEs from the data-driven multiparametric meta-regression analysis model (effects of HPV16/18-AS04 on CIN3+ caused by HPV16/18 types), adjusted for age and analytical cohort (analysis 1). CI = confidence interval; CIN3+ =grade 3 cervical intraepithelial neoplasia or worse; HPV = human papillomavirus; TVC = total vaccinated cohort; VE = vaccine effect. Note for interpretation of graphs: Subgroups. 1 = Lehtinen 2012,^20^ age at first vaccination 15–17 years, TVC, time since vaccination 0–4 years. 2 = Lehtinen 2012,^20^ age at first vaccination 18–20 years, TVC, time since vaccination 0–4 years. 3 = Lehtinen 2012,^20^ age at first vaccination 21–25 years, TVC, time since vaccination 0–4 years. 4 = Lehtinen 2012,^20^ age at first vaccination 15–17 years, TVC-naïve, time since vaccination 0–4 years. 5 = Lehtinen 2012,^20^ age at first vaccination 18–20 years, TVC-naïve, time since vaccination 0–4 years. 6 = Lehtinen 2012,^20^ age at first vaccination 21–25 years, TVC-naïve, time since vaccination 0–4 years. 7 = Porras 2020,^22^ age at first vaccination 18–25 years, TVC-naïve, time since vaccination 0–4 years. 8 = Shing 2022,^23^ age at first vaccination 18–25 years, TVC, time since vaccination 7–11 years. 9 = Rebolj 2022,^25^ age at first vaccination 14–17 years, TVC, time since vaccination 7–11 years. The dotted line represents the 95% CI. Purple and blue curves represent the predicted VE as a function of age for TVC and TVC-naïve populations, respectively. The centre of pink and blue bubbles represents the observed VEs in TVC and TVC-naïve populations, respectively. Size of the bubbles is proportional to the inverse of the variance (i.e. the weight in a classical meta-analysis). The numbers on the bubbles represent each subgroup, with details about the age at first vaccination or the age range at the time of vaccination provided in the figure legend.

In the multiparametric meta-regression analysis, all predictor combinations were evaluated and compared using AIC to select a final model, which included ‘age at first vaccination’ and ‘analytical cohort.’ After adjusting for analytical cohort (TVC *vs* TVC-naïve), ‘age at first vaccination’ emerged as the most impactful variable on the outcome (*P* = .01; Supplementary Table S7). Interestingly, ‘time since vaccination’ was not a key explanatory factor, suggesting that vaccine’s effect persists over time. Figure 1 shows predictions from the selected model, adjusting for ‘age at first vaccination’ and ‘analytical cohort,’ indicating a higher effect for TVC-naïve and for younger age at vaccination. The proportion of total variance due to study heterogeneity (*I^2^*) was 46%; between-study variability (*σ*^2^) was 0.23; and the proportion of heterogeneity explained by the model (*R*^2^) was 62%^29^ (Supplementary Table S7). Incorporating covariates into the meta-regression reduced study variability and unaccounted heterogeneity compared with the simple meta-analysis (*σ^2^* = 0.62, *I^2^* = 75%) (Supplementary Fig. S4).

**Figure 2:**
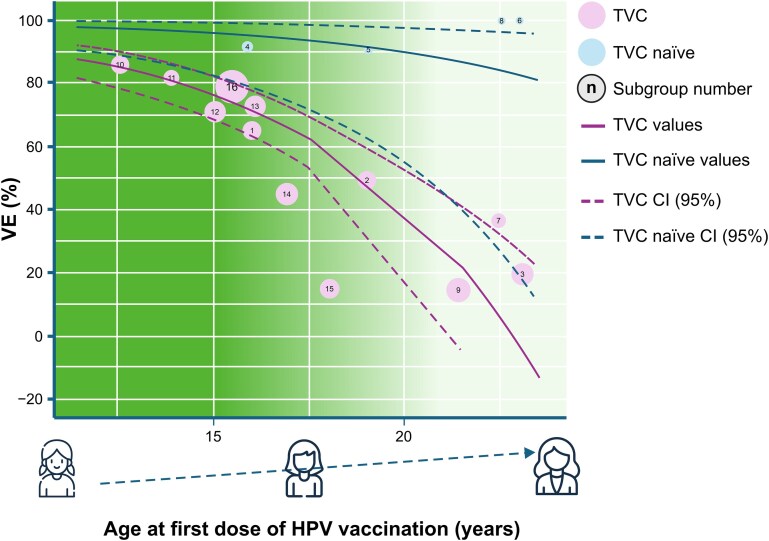
Predicted VEs from the data-driven multiparametric meta-regression analysis model (effects of HPV16/18-AS04 on CIN3+, irrespective of HPV type), adjusted for age and analytical cohort (analysis 2). CI = confidence interval; CIN3+ =grade 3 cervical intraepithelial neoplasia or worse; HPV = human papillomavirus; TVC = total vaccinated cohort; VE = vaccine effect. Note for interpretation of graphs: Subgroup: 1 = Lehtinen 2012,^20^ age at first vaccination 15–17 years, TVC, time since vaccination 0–4 years. 2 = Lehtinen 2012,^20^ age at first vaccination 18–20 years, TVC, time since vaccination 0–4 years. 3 = Lehtinen 2012,^20^ age at first vaccination 21–25 years, TVC, time since vaccination 0–4 years. 4 = Lehtinen 2012,^20^ age at first vaccination 15–17 years, TVC-naïve, time since vaccination 0–4 years. 5 = Lehtinen 2012,^20^ age at first vaccination 18–20 years, TVC-naïve, time since vaccination 0–4 years. 6 = Lehtinen 2012,^20^ age at first vaccination 21–25 years, TVC-naïve, time since vaccination 0–4 years. 7 = Konno 2014,^21^ age at first vaccination 20–25 years, TVC, time since vaccination 0–4 years. 8 = Konno 2014,^21^ age at first vaccination 20–25 years, TVC-naïve, time since vaccination 0–4 years. 9 = Shing 2022,^23^ age at first vaccination 18–25 years, TVC, time since vaccination 7–11 years. 10 = Palmer 2019,^16^ age at first vaccination 12–13 years, TVC, time since vaccination 0–8 years. 11 = Palmer 2019,^16^ age at first vaccination 14 years, TVC, time since vaccination 0–6 years. 12 = Palmer 2019,^16^ age at first vaccination 15 years, TVC, time since vaccination 0–5 years. 13 = Palmer 2019,^16^ age at first vaccination 16 years, TVC, time since vaccination 0–4 years. 14 = Palmer 2019,^16^ age at first vaccination 17 years, TVC, time since vaccination 0–3 years. 15 = Palmer 2019,^16^ age at first vaccination ≥18 years, TVC, time since vaccination 0–2 years. 16 = Rebolj 2022,^25^ age at first vaccination 14–17 years, TVC, time since vaccination 7–11 years. The dotted line represents the 95% CI. Purple and blue curves represent the predicted VE as a function of age for TVC and TVC-naïve populations, respectively. The centre of pink and blue bubbles represents the observed VEs in TVC and TVC-naïve populations, respectively. Size of the bubbles is proportional to the inverse of the variance (i.e. the weight in a classical meta-analysis). The numbers on the bubbles represent each subgroup, with details about the age at first vaccination or the age range at the time of vaccination provided in the figure legend.

VEs of HPV16/18-AS04 on CIN3+, caused by HPV 16/18 or irrespective of HPV type, from RCTs and observational studies combined were also considered (analyses 1 and 2 in Table 2; Figs. 1 and 2, respectively; Supplementary Figs. S5―S8; and results of secondary analyses). Separate analyses of RCTs and observational studies were subsequently performed (analyses 3―6 in Table 2; Supplementary Figs. S9―S12; and results of secondary analyses).

This approach allowed exploration of variations in VE dependent on the study design and endpoints (Table 2 and Supplementary material).

## Discussion

### Main findings of this study

This systematic review and quantitative analysis demonstrate that the HPV16/18-AS04 vaccine effectively prevents advanced cervical premalignant lesions and cervical cancer in adolescent girls and women vaccinated at age 12–25 years. This finding is consistent across RCT follow-ups, real-world observational studies and their combinations. Pooled VEs ranged within 48%–78%, regardless of HPV DNA classification (Table 2). Variations in VEs are most likely attributable to differences in subgroup age and time since vaccination. Multiparametric meta-regression results (analyses 1 and 2) across study designs consistently show that the highest VEs occur in the youngest age groups at vaccination, whether considering VEs on CIN3+ caused by vaccine HPV types or any HPV type. This impact of age at first vaccination is particularly pronounced in the TVC (Figs. 1–2). To our knowledge, this is the first evaluation of long-term effects of HPV16/18-AS04 vaccine (4 years for RCTs and 10–11 years for observational studies) against CIN3+ and cervical cancer, including real-world data.

Greater VEs (vaccine efficacy and/or effectiveness) of HPV16/18-AS04 were observed in the youngest age groups assessed, with many studies reporting reduced VEs among recipients who initiated vaccination at a later age. This is likely due to the vaccine administration before HPV exposure, which typically occurs through sexual activity.^30^ Larger VEs and a smaller impact of age at vaccination (Figs. 1–2) were observed in studies wherein the analytical cohort included women who were HPV DNA-negative at enrolment (TVC-naïve, Supplementary Table S3). This confirms findings from pivotal clinical trials demonstrating higher efficacy when the vaccine is administered before HPV exposure.^31^^,^^32^ This trend was also evident in population-based studies (Palmer, 2019)^18^ wherein adolescents vaccinated at 17 years were more than thrice as likely to be diagnosed with CIN3+ compared with those vaccinated at 12–13 years (OR = 0.55 [95% CI 0.36–0.83] *vs* OR = 0.14 [95% CI 0.08–0.25], respectively).

This systematic review also revealed long-term, broad protection conferred by HPV16/18-AS04 against CIN3+ caused by non-vaccine types based on both RCT follow-ups^21^ and observational^18^^,^^27^ studies.

Long-term immunogenicity of HPV16/18-AS04 was demonstrated in adolescents vaccinated at 16–17 years, with high neutralizing antibody maintained up to 12 years post-vaccination across all age strata (18–45 years) and levels 6- to 12-fold higher than those induced by Gardasil (Merck Sharp & Dohme LLC, Rahway, NJ, USA).^33^ Studies indicate that the adjuvant in HPV16/18-AS04 elicits a strong, broad and durable immune response.^34^ Although the clinical implications of these findings were uncertain, our analysis estimated consistent HPV16/18-AS04 VEs in 4-year RCT follow-ups and up to over 10–11 years in observational and population-based studies. These long-term effects translated into a reduction, and in some settings, near elimination of cervical cancer after HPV16/18-AS04 NIP implementation.^26^^,^^27^ Recent reports from the Netherlands^35^ showed a vaccine effectiveness of 95.8% against persistent HPV16/18 infection and 64.6% against persistent cross-protective type infections (HPV31/33/45) 10 years post-vaccination, with no signs of waning immunity. Similarly, in Scotland,^36^ no cases of invasive cervical cancer were reported among women immunized with HPV16/18-AS04 at age 12–13 years, irrespective of the number of doses received. Long-term seropositivity is typically higher among those vaccinated at younger ages compared with older groups, particularly those aged ≥25 years.^37^ Our meta-regression analysis revealed that time since vaccination impacts vaccine effectiveness, irrespective of HPV type, when pooling data from observational studies (Table 2).

This review also revealed effects of HPV16/18-AS04 on other endpoints (CIN3, adenocarcinoma in situ [AIS], cervical cancer), herd effects and cross-protection. Limited data allowed for only a narrative review (Supplementary material).

Vaccine effectiveness is affected by vaccine efficacy, vaccination policies and real-world administration conditions. Variations in age recommendations impact effectiveness, with vaccination at a younger age proving more effective. These findings should compel policymakers and the broader community to prioritize HPV vaccination at the earliest recommended age, confident that early immunization provides the most durable and potent protection.

### What is already known on this topic

Evidence of vaccine efficacy against CIN3+ caused by vaccine HPV types has been reported in the according-to-protocol for efficacy cohort (ATP-E cohort) up to 40 months post–dose 3 and in the TVC up to 44 months post–dose 1 irrespective of HPV DNA type present in the lesion, in women vaccinated at ages 15–25 years.^36^ Data on vaccine effectiveness against CIN3+ from post-hoc RCTs and retrospective population-based studies have also been published.^36^ However, our search revealed a lack of long-term global estimates of VEs and impactful variables.

### What this study adds

To our knowledge, our systematic review and meta-regression study is the first to provide globally aggregated data on the long-term protection offered by the HPV16/18-AS04 vaccine against CIN3+, the closest surrogate endpoint for cervical cancer risk. This study incorporates results from long-term follow-ups of RCTs and observational studies (including nationwide surveillance data), analysing data both combined and independently from women who were HPV-naïve at vaccination or included irrespective of baseline HPV status and those vaccinated with ≥1 vaccine dose (with ≥75% receiving three doses). Additionally, our study provides a descriptive synthesis of other VEs, such as herd effects and cross-protection, on CIN3+ and other endpoints such as CIN3, AIS and cervical cancer. The results emphasize the sustained long-term protection conferred by HPV16/18-AS04, particularly when administered at a younger age, regardless of the HPV type detected in the lesion.

Determining global estimates of the long-term protection conferred by the HPV16/18-AS04 vaccine against advanced premalignant cervical lesions and cancer and identifying impactful variables (e.g. early vaccination age) is crucial for developing adequate public health policies. These data should enable policymakers to plan interventions promoting early HPV vaccination, aligning with the WHO’s goals for eliminating cervical cancer.

### Limitations and strengths of this study

As with systematic literature reviews, this study has limitations in retrieving all available information. Several studies, particularly observational studies, carried some risk of bias due to their design. However, all studies acknowledged their limitations and used different methods to address bias and confounding, ensuring robust conclusions. The meta-regression analysis included only few studies (*n* = 7), potentially affecting statistical power. Heterogeneity was observed; however, we applied quality appraisal tools, fitted random-effects models and used data-driven (AIC) selection to address it. We considered several scenarios (analyses 1–6) to evaluate the robustness of the results. The identified covariates explained a substantial proportion of the heterogeneity, improving predictions for decision-making. This study’s strengths include its wide geographic scope; inclusion of RCT follow-ups and broad population-based studies spanning several years; and the assessment of varied age groups at vaccination, allowing a thorough evaluation of VEs on CIN3+, the closest surrogate endpoint for cervical cancer risk.^38^

## Conclusions

The data-driven multiparametric meta-regression graphs for the combined analyses of RCTs and observational studies clearly illustrate the key findings of this systematic review: VEs are largest when vaccination occurs at a younger age, irrespective of study design and HPV type. Delaying HPV vaccination results in a missed opportunity for optimal protection that cannot be regained later. Public health strategies should prioritize early vaccination as the cornerstone of HPV prevention efforts. This approach will maximize vaccine effectiveness, ensure widespread coverage and provide robust, long-lasting immunity, leveraging the full potential of HPV vaccination programmes and moving decisively towards the global elimination of cervical cancer.

## Supplementary Material

DV-014882_Cervarix_SLR_MS_Online_Suppl_Final_Clean_Updated_fdag002

## Data Availability

The data supporting this study are available from the corresponding author upon reasonable request.

## HPV16/18-AS04 vaccine (Cervarix^®^) trademark

HPV16/18-AS04 vaccine (Cervarix^®^) is a trademark owned by or licensed to GSK.

## Notes

**Adoración Navarro-Torné**, Director

**Emmanuel Aris**, Director

**Andrea Callegaro**, Director

**Bernd Benninghoff**, CEO

**Huifeng Yun**, Senior Director and Head

**Volker Vetter**,
