# Supplementary material for: Maximizing impact: the power of early HPV vaccination for long-term protection―lessons from a systematic review and meta-regression analysis
Source: J Public Health (Oxf). 2026 Jan 30;48(1):216–27. doi: 10.1093/pubmed/fdag002 (PMC13017608; doi:10.1093/pubmed/fdag002)
Supplement: DV-014882_Cervarix_SLR_MS_Online_Suppl_Final_Clean_Updated_fdag002 [file dv-014882_cervarix_slr_ms_online_suppl_final_clean_updated_fdag002.docx]

**Supplementary material**

**Search terms and strings**

The literature search was based on the following concepts articulating different search terms:

**Concept 1**

“papillomavirus vaccine”, “papillomavirus vaccination”, “HPV vaccine”, “HPV vaccination”, “CERVARIX”, “bivalent human papillomavirus vaccine”.

**Concept 2**

“program* evaluation”, “population surveillance”, “sentinel surveillance”, “vaccine effectiveness”, “vaccine efficacy”.

**Concept 3**

“high-grade cervical intraepithelial neoplasia”, “cervical intraepithelial neoplasia grade 3”, “high-grade CIN”, “cervical severe dysplasia”, “cervical severe dyskaryosis”, “cervical carcinoma *in-situ*” “uterine cervical neoplasm”, “CIN 3”, “cervical invasive carcinoma”, “high-grade squamous intraepithelial lesion”, “HSIL”.

Included MeSH and Emtree terms when available. Hand-search of references of relevant papers were also performed.

Note: Asterisk (*) is a truncation symbol to broaden results in the selected databases that allows to look for variations of words. They can be used in a keyword search to retrieve alternate word endings (program* will retrieve “program”, “programs”, “programme”, “programmes”, etc).

**Table SI: Search terms for the different databases**

| Keyword | MeSH (PubMed) | Emtree (EMBASE) | Scopus |
| --- | --- | --- | --- |
| papillomavirus vaccine | “Papillomavirus Vaccines” [MeSH] | ‘Human papilloma virus vaccine’/exp | papillomavirus vaccine |
| papillomavirus vaccination | No MeSH term | No Emtree term | papillomavirus vaccination |
| HPV vaccine | “Papillomavirus Vaccines” [MeSH] | No Emtree term | HPV vaccine |
| HPV vaccination | “Papillomavirus Vaccines” [MeSH] | ‘hpv vaccination’/exp | HPV vaccination |
| CERVARIX | No MeSH term | No Emtree term | CERVARIX |
| bivalent human papillomavirus vaccine | No MeSH term | ‘bivalent human papillomavirus vaccine’ | “bivalent human papillomavirus vaccine” |
| program* evaluation | “Program Evaluation” [MeSH] | ‘program evaluation’/exp | “program* evaluation” |
| population surveillance | “Population Surveillance” [MeSH] | ‘population surveillance’/exp | “population surveillance” |
| sentinel surveillance | “Sentinel Surveillance” [MeSH] | ‘sentinel surveillance’/exp | “sentinel surveillance” |
| vaccine efficacy | “Vaccine Efficacy” [MeSH] | ‘vaccine efficacy’ | “vaccine efficacy” |
| vaccine effectiveness | “Vaccine Efficacy” [MeSH] | ‘vaccine effectiveness’/exp | “vaccine effectiveness” |
| cervical intraepithelial neoplasia grade 3 | “Cervical Intraepithelial Neoplasia” [MeSH]  “Uterine Cervical Neoplasms” [MeSH] | ‘cervical intraepitelial neoplasia 3’/exp | cervical intraepithelial neoplasia |
| cervical severe dysplasia | “Cervical Intraepithelial Neoplasia” [MeSH]  “Uterine Cervical Dysplasia” [MeSH] | ‘uterine cervix dysplasia’/exp | cervical dysplasia |
| cervical severe dyskaryosis | “Cervical Intraepithelial Neoplasia” [MeSH] | No Emtree term | cervical severe dyskaryosis |
| uterine cervical neoplasm | “Uterine Cervical Neoplasms” [MeSH] | ‘uterine cervix cancer’/exp | uterine cervical neoplasm |
| uterine cervical carcinoma | No MeSH term | uterine cervical carcinoma | uterine cervix carcinoma |
| high-grade CIN | No MeSH term | No Emtree term | high-grade CIN |
| high-grade squamous intraepithelial lesion | “Squamous Intraepithelial Lesions” [MeSH] | ‘high-grade squamous intraepithelial lesion of the cervix’/exp | high-grade squamous intraepithelial lesion |
| high-grade cervical intraepithelial neoplasia | No MeSH term | ‘uterine cervix carcinoma in situ’/exp | high-grade cervical intraepithelial neoplasia |
| cervical carcinoma in-situ | “Cervical Intraepithelial Neoplasia” [MeSH] | ‘uterine cervix carcinoma in situ’/exp | cervical carcinoma in-situ |
| CIN 3 | “Cervical Intraepithelial Neoplasia” [MeSH] | No Emtree term | CIN 3 |
| cervical invasive carcinoma/cancer | No MeSH term | ‘uterine cervix cancer’/exp | cervical invasive carcinoma/cancer |
| HSIL | “Cervical Intraepithelial Neoplasia” [MeSH] | No Emtree term | HSIL |

CIN=cervical intraepithelial neoplasm. HSIL= high grade squamous intraepithelial lesion. RCTs=randomised controlled trials. VE= vaccine effects.

For this study, vaccine efficacy refers to controlled conditions (RCTs), vaccine effectiveness refers to real-world conditions (observational studies), and VE is a combination of vaccine efficacy and vaccine effectiveness.

**Search strategy**

**PubMed**:

"Papillomavirus Vaccines" [MeSH] OR "papilloma virus vaccin*"[tw] OR "papillomavirus vaccin*"[tw] OR "hpv vaccin*"[tw] OR CERVARIX[tw] AND (vaccine[tw] AND (efficacy[tw] OR effectiveness[tw])) OR ("program* evaluation*"[tw] OR "Program Evaluation"[MeSH] OR "population surveillance"[tw] OR "Population Surveillance"[MeSH] OR "sentinel surveillance"[tw] OR "Sentinel Surveillance"[MeSH] OR "Vaccine Efficacy"[MeSH]) AND "Uterine cervical dysplasia"[MeSH] OR "Squamous intraepithelial lesions"[MeSH] OR "Cervical intraepithelial neoplasia"[MeSH] OR "Uterine cervical neoplasms"[MeSH] OR "uterine cervical carcinoma*"[tw] OR "uterine cervix carcinoma*"[tw] OR "cervical invasive carcinoma"[tw] OR "cervical invasive cancer"[tw] OR "high-grade CIN"[tw] OR "cervical intraepithelial neoplas*"[tw] OR "CIN 3"’[tw] OR ‘HSIL’[tw] OR ((cervical[tw] OR cervical[tw]) AND (dysplasia*[tw] OR dyskaryosis[tw])) AND (Humans[MeSH] OR human*[tw]) NOT review[Publication Type] AND (“2000/01/01”[Date - Publication]:”2022/06/22”[Date - Publication]).

Filters:

Humans.

from 01/01/2000 to 21/06/2022.

Not conference abstract, conference paper, or review.

**EMBASE**:

(('wart virus vaccin*' OR 'papillomavirus vaccin*' OR 'papilloma virus vaccin*' OR 'hpv vaccin*' OR CERVARIX ):ti,ab,kw OR 'hpv vaccination'/exp OR ' Human papilloma virus vaccine'/exp) AND 'vaccine effectiveness'/exp OR 'drug efficacy'/exp OR 'sentinel surveillance'/exp OR 'program evaluation'/exp OR 'population surveillance'/exp OR 'program* evaluation':ti,ab,kw OR ((population NEAR/5 surveillance):ti,ab,kw) OR 'sentinel surveillance':ti,ab,kw OR ((vaccine NEAR/5 efficacy):ti,ab,kw) AND 'cervical intraepithelial neoplasia 3'/exp OR 'uterine cervix dysplasia'/exp OR 'uterine cervix tumor'/exp OR 'uterine cervix cancer'/exp OR 'high grade squamous intraepithelial lesion of the cervix'/exp OR 'uterine cervix carcinoma in situ'/exp OR ('uterine cervix carcinoma*' OR 'high-grade CIN' OR 'CIN 3' OR 'HSIL' OR cervical near/3 dysplasia* OR cervix near/3 dysplasia* OR 'cervical severe dyskaryosis'):ti,ab,kw AND [humans]/lim NOT 'conference abstract'/it OR 'conference paper'/it OR 'review'/it AND [01-05-2000]/sd NOT [22-06-2022]/sd.

Filters:

Humans.

From 01/01/2000 to 21/06/2022.

Not conference abstract, conference paper, or review.

Note: At the time of the preparation of the manuscript we realised that “dyskariosis” was used for the search in EMBASE instead of the correct spelling “dyskaryosis”. Post-hoc searches in EMBASE both for 'cervical severe dyskariosis' and 'cervical severe dyskaryosis' search strings resulted in “0” hits retrieved in both cases. Therefore, the final output is not affected by this typo.

**Scopus**:

((((TITLE-ABS-KEY (("wart virus vaccin*" OR "papillomavirus vaccin*" OR "papilloma virus vaccin*" OR "hpv vaccin*" OR CERVARIX) AND ((vaccine or program*)w/5 (efficacy OR effectiveness or impact)) OR ("population surveillance" OR "sentinel surveillance") AND (("cervical intraepithelial neoplasia" w/2 3) or ("uterine cervi*" w/1 (carcinoma* or neoplasm*)) or "high-grade CIN" or "CIN 3" or "high-grade squamous intraepithelial lesion*" or "HSIL" or ((cervical or cervical) w/2 (dysplasia* or dyskaryosis or "invasive carcinoma")))))) AND (TITLE-ABS-KEY ((human*)))) AND NOT (((DOCTYPE (re) OR DOCTYPE (cp)))) AND (PUBYEAR > 1999).

Filters:

Title, Abstract, Keywords.

Humans.

From 01/01/2000 to 21/06/2022.

Not conference abstract, conference paper, or review.

**Cochrane CENTRAL**:

(CERVARIX): ti,ab,kw OR (bivalent human papillomavirus vaccine):ti,ab,kw

Filters:

Content type: Trials.

Cochrane Library publication date: Between Jan 2000 and Jun 2022.

CENTRAL Trials only original publication year: All years.

Search word variations.

**List of full papers assessed for inclusion**

1. Acuti Martellucci C, Nomura S, Yoneoka D. *et al*. Human papillomavirus vaccine effectiveness within a cervical cancer screening programme: cohort study. *BJOG* 2021;**128**:532–39.
2. Apter D, Wheeler CM, Paavonen J. *et al*. Efficacy of human papillomavirus 16 and 18 (HPV-16/18) AS04-adjuvanted vaccine against cervical infection and precancer in young women: Final event-driven analysis of the randomized, double-blind PATRICIA trial. *Clin Vaccine Immunol* 2015;**22**:36173.
3. Arbyn M, Broeck DV, Benoy I. *et al*. Surveillance of effects of HPV vaccination in Belgium. *Cancer Epidemiol* 2016;**41**:152–8.
4. Beachler DC, Kreimer AR, Schiffman M. *et al*. Multisite HPV16/18 vaccine efficacy against cervical, anal, and oral HPV infection. *J Natl Cancer Inst* 2016;**108**:djv302.
5. Brotherton JML, Tabrizi SN, Garland SM. Does HPV type 16 or 18 prevalence in cervical intraepithelial neoplasia grade 3 lesions vary by age? An important issue for postvaccination surveillance. *Future Microbiol* 2012;**7**:193–99.
6. Brown DR, Kjaer SK, Sigurdsson K. *et al*. The impact of quadrivalent human papillomavirus (HPV; types 6, 11, 16, and 18) L1 virus-like particle vaccine on infection and disease due to oncogenic nonvaccine HPV types in generally HPV-naive women aged 16-26 years. *J Infect Dis* 2009;**199**:926–35.
7. Cameron RL, Kavanagh K, Cameron Watt D. *et al*. The impact of bivalent HPV vaccine on cervical intraepithelial neoplasia by deprivation in Scotland: reducing the gap. *J Epidemiol Community Health* 2017;**71**:954–60.
8. Cameron RL, Pollock KG. The impact of the human papillomavirus vaccine in Scotland: A changing landscape. *Clinical Pharmacist* 2017b.3;**9**(3).
9. Casajuana-Pérez A, Ramírez-Mena M, Ruipérez-Pacheco E. *et al*. Effectiveness of prophylactic human papillomavirus vaccine in the prevention of recurrence in women conized for HSIL/CIN 2-3: The VENUS study. *Vaccines (Basel)* 2022;**10**:288.
10. Chen G, Zheng P, Gao L. *et al*. Prevalence and genotype distribution of human papillomavirus in women with cervical cancer or cervical intraepithelial neoplasia in Henan province, central China. *J Med Virol* 2020;**92**:3743–49.
11. Clark M, Jembere N, Kupets R. The impact of a universal human papilloma virus (HPV) vaccination program on lower genital tract dysplasia and genital warts. *Prev Med*. 2021;150:106641.
12. De Carvalho N, Teixeira J, Roteli-Martins CM. *et al*. Sustained efficacy and immunogenicity of the HPV-16/18 AS04-adjuvanted vaccine up to 7.3 years in young adult women. *Vaccine* 2010;**28**:6247–55.
13. Del Mistro A, Battagello J, Weis L. *et al*. A retrospective cohort study of young women spontaneously choosing to be vaccinated against HPV: Outcomes from their first cervical cancer screening test. *Viruses* 2021;**13**:486.
14. Donken R, van Niekerk D, Hamm J. *et al*. Declining rates of cervical intraepithelial neoplasia in British Columbia, Canada: An ecological analysis on the effects of the school-based human papillomavirus vaccination program. *Int J Cancer* 2021;**149**:191–99.
15. Falcaro M, Castañon A, Ndlela B. *et al*. The effects of the national HPV vaccination programme in England, UK, on cervical cancer and grade 3 cervical intraepithelial neoplasia incidence: a register-based observational study. *Lancet* 2021;**398**:2084–92.
16. Hallowell BD, Saraiya M, Thompson TD. *et al*. Population-based assessment of HPV genotype-specific cervical cancer survival: CDC Cancer Registry Sentinel Surveillance System. *JNCI Cancer Spectr* 2018;**2**:pky036.
17. Harari A, Chen Z, Rodríguez AC. *et al*. Crossprotection of the bivalent human papillomavirus (HPV) vaccine against variants of genetically related high-risk HPV infections. *J Infect Dis* 2016;**213**:939–47.
18. Hariri S, Bennett NM, Niccolai LM. *et al*. Reduction in HPV 16/18-associated high grade cervical lesions following HPV vaccine introduction in the United States - 2008-2012. *Vaccine* 2015;**33**:1608–13.
19. Harper DM, Franco EL, Wheeler CM. *et al*. Sustained efficacy up to 4.5 years of a bivalent L1 virus-like particle vaccine against human papillomavirus types 16 and 18: follow-up from a randomised control trial. *Lancet* 2006;**367**:1247–55.
20. Hildesheim A, Wacholder S, Catteau G. *et al*. Efficacy of the HPV-16/18 vaccine: final according to protocol results from the blinded phase of the randomized Costa Rica HPV-16/18 vaccine trial. *Vaccine* 2014;**32**:5087–97.
21. Hiramatsu K, Ueda Y, Yagi A. *et al*. The efficacy of human papillomavirus vaccination in young Japanese girls: the interim results of the OCEAN study. *Hum Vaccin Immunother* 2022;**18**:1951098.
22. Ikeda S, Ueda Y, Hara M. *et al*. Human papillomavirus vaccine to prevent cervical intraepithelial neoplasia in Japan: A nationwide case-control study. *Cancer Sci* 2021;**112**:839–46.
23. Johnson Jones ML, Gargano JW, Powell M. *et al*. Effectiveness of 1, 2, and 3 doses of human papillomavirus vaccine against high-grade cervical lesions positive for human papillomavirus 16 or 18. *Am J Epidemiol* 2020;**189**:265–76.
24. Khatun S, Akram Hussain SM, Chowdhury S. *et al*. Safety and immunogenicity profile of human papillomavirus-16/18 AS04 adjuvant cervical cancer vaccine: a randomized controlled trial in healthy adolescent girls of Bangladesh. *Jpn J Clin Oncol* 2012;**42**:36–41.
25. Kjaer SK, Dehlendorff C, Belmonte F. *et al*. Real-world effectiveness of human papillomavirus vaccination against cervical cancer. *J Natl Cancer Inst* 2021;**113**:1329–35.
26. Konno R, Tamura S, Dobbelaere K. *et al*. Efficacy of human papillomavirus 16/18 AS04-adjuvanted vaccine in Japanese women aged 20 to 25 years: Interim analysis of a phase 2 double-blind, randomized, controlled trial. *Int J Gynecol Cancer* 2010;**20**:404–10.
27. Konno R, Yoshikawa H, Okutani M. *et al*. Efficacy of the human papillomavirus (HPV)-16/18 AS04-adjuvanted vaccine against cervical intraepithelial neoplasia and cervical infection in young Japanese women. *Hum Vaccin Immunother* 2014;**10**:1781–94.
28. Konno R, Konishi H, Sauvaget C, Ohashi Y, Kakizoe T. Effectiveness of HPV vaccination against high grade cervical lesions in Japan. *Vaccine* 2018;**36**:7913–15.
29. Lehtinen M, Paavonen J, Wheeler CM. *et al*. Overall efficacy of HPV-16/18 AS04adjuvanted vaccine against grade 3 or greater cervical intraepithelial neoplasia: 4-year end-of-study analysis of the randomised, doubleblind PATRICIA trial. *Lancet Oncol* 2012;**13**:89–99.
30. Lehtinen M, Lagheden C, Luostarinen T. *et al*. Ten-year follow-up of human papillomavirus vaccine efficacy against the most stringent cervical neoplasia end-point - registry-based follow-up of three cohorts from randomized trials. *BMJ Open* 2017;**7**:e015867.
31. Naud PS, Roteli-Martins CM, De Carvalho NS. *et al*. Sustained efficacy, immunogenicity, and safety of the HPV-16/18 AS04-adjuvanted vaccine: final analysis of a long-term follow-up study up to 9.4 years post-vaccination. *Hum Vaccin Immunother* 2014;**10**:2147–62.
32. Onuki M, Yamamoto K, Yahata H. *et al*. Human papillomavirus vaccine effectiveness by age at first vaccination among Japanese women. *Cancer Science* 2022;**113**:1428–34.
33. Paavonen J, Naud P, Salmerón J. *et al*. Efficacy of human papillomavirus (HPV)-16/18 AS04-adjuvanted vaccine against cervical infection and precancer caused by oncogenic HPV types (PATRICIA): final analysis of a doubleblind, randomised study in young women. *Lancet* 2009;**374**:301–14.
34. Palmer T, Wallace L, Pollock KG. *et al*. Prevalence of cervical disease at age 20 after immunization with bivalent HPV vaccine at age 12-13 in Scotland: retrospective population study. *BMJ* 2019;**365**:1161.
35. Porras C, Tsang SH, Herrero R. *et al*. Efficacy of the bivalent HPV vaccine against HPV 16/18-associated precancer: long-term follow-up results from the Costa Rica Vaccine Trial. *Lancet Oncol* 2020;**21**:1643–52.
36. Powell SE, Hariri S, Steinau M. *et al*. Impact of human papillomavirus (HPV) vaccination on HPV 16/18-related prevalence in precancerous cervical lesions. *Vaccine* 2012;**31**:109–13.
37. Racey CS, Albert A, Donken R. *et al*. Cervical Intraepithelial neoplasia rates in British Columbia women: A population-level data linkage evaluation of the school-based HPV immunization program. *J Infect Dis* 2020;**221**:81–90.
38. Rana MM, Huhtala H, Apter D. *et al*. Understanding long-term protection of human papillomavirus vaccination against cervical carcinoma: Cancer registry-based follow-up. *Int J Cancer* 2013;**132**:2833–38.
39. Rebolj M, Pesola F, Mathews C. *et al*. The impact of catchup bivalent human papillomavirus vaccination on cervical screening outcomes: an observational study from the English HPV primary screening pilot. *Br J Cancer* 2022;**127**:278–87.
40. Romanowski B, de Borba PC, Naud PS. *et al*. Sustained efficacy and immunogenicity of the human papillomavirus (HPV)16/18 AS04-adjuvanted vaccine: analysis of a randomised placebo-controlled trial up to 6.4 years. *Lancet* 2009;**374**:1975–85.
41. Roteli-Martins CM, Naud P, De Borba P. *et al*. Sustained immunogenicity and efficacy of the HPV-16/18 AS04-adjuvanted vaccine: up to 8.4 years of follow-up. *Hum Vaccin Immunother* 2012;**8**:390–97.
42. Ryser M, Berlaimont V, Karkada N. *et al*. Post-hoc analysis from phase III trials of human papillomavirus vaccines: considerations on impact on non-vaccine types. *Expert Rev Vaccines* 2019;**18**:309–22.
43. Shiko Y, Konno R, Konishi H. *et al*. Effectiveness of HPV vaccination against the development of high-grade cervical lesions in young Japanese women. *BMC Infect Dis* 2020;**20**:808.
44. Shing JZ, Hu S, Herrero R. *et al*. Precancerous cervical lesions caused by non-vaccine-preventable HPV types after vaccination with the bivalent AS04-adjuvanted HPV vaccine: an analysis of the long-term follow-up study from the randomised Costa Rica HPV Vaccine Trial. *Lancet Oncol* 2022;**23**:940–49.
45. Silverberg MJ, Leyden WA, Lam JO. *et al*. Effectiveness of 'catch-up' human papillomavirus vaccination to prevent cervical neoplasia in immunosuppressed and non-immunosuppressed women. *Vaccine*. 2020;**38**:4520–23.
46. Skinner SR, Szarewski A, Romanowski B. *et al*. Efficacy, safety, and immunogenicity of the human papillomavirus 16/18 AS04-adjuvanted vaccine in women older than 25 years: 4-year interim follow-up of the phase 3, double-blind, randomised controlled VIVIANE study. *Lancet* 2014;**384**:2213–27.
47. Skinner SR, Wheeler CM, Romanowski B. *et al*. Progression of HPV infection to detectable cervical lesions or clearance in adult women: Analysis of the control arm of the VIVIANE study. *Int J Cancer* 2016;**138**:2428–38.
48. Szarewski A, Poppe WA, Skinner SR. *et al*. Efficacy of the human papillomavirus (HPV)-16/18 AS04-adjuvanted vaccine in women aged 15-25 years with and without serological evidence of previous exposure to HPV-16/18. *Int J Cancer* 2012;**131**:106–16.
49. Tota JE, Struyf F, Sampson JN. *et al*. Efficacy of the AS04-Adjuvanted HPV16/18 vaccine: Pooled analysis of the costa rica vaccine and PATRICIA randomized controlled trials. *J Natl Cancer Inst* 2020;**112**:818–28.
50. Tota JE, Struyf F, Hildesheim A. *et al*. Efficacy of AS04-adjuvanted vaccine against human papillomavirus (HPV) types 16 and 18 in clearing incident HPV infections: Pooled analysis of data from the Costa Rica Vaccine Trial and the PATRICIA study. *J Infect Dis* 2021;**223**:1576–81.
51. Tozawa-Ono A, Kamada M, Teramoto K. *et al*. Effectiveness of human papillomavirus vaccination in young Japanese women: a retrospective multi-municipality study. *Hum Vaccin Immunother* 2021;**17**:950–54.
52. Wheeler CM, Castellsagué X, Garland SM. *et al*. Cross-protective efficacy of HPV-16/18 AS04-adjuvanted vaccine against cervical infection and precancer caused by non-vaccine oncogenic HPV types: 4-year end-of-study analysis of the randomised, double-blind PATRICIA trial. *Lancet Oncol* 2012;**13**:100–10.
53. Yagi A, Ueda Y, Nakagawa S. *et al*. A nationwide birth year-by-year analysis of effectiveness of HPV vaccine in Japan. *Cancer Sci* 2021;**112**:3691–98.

Fig. S1: PRISMA flow diagram

*
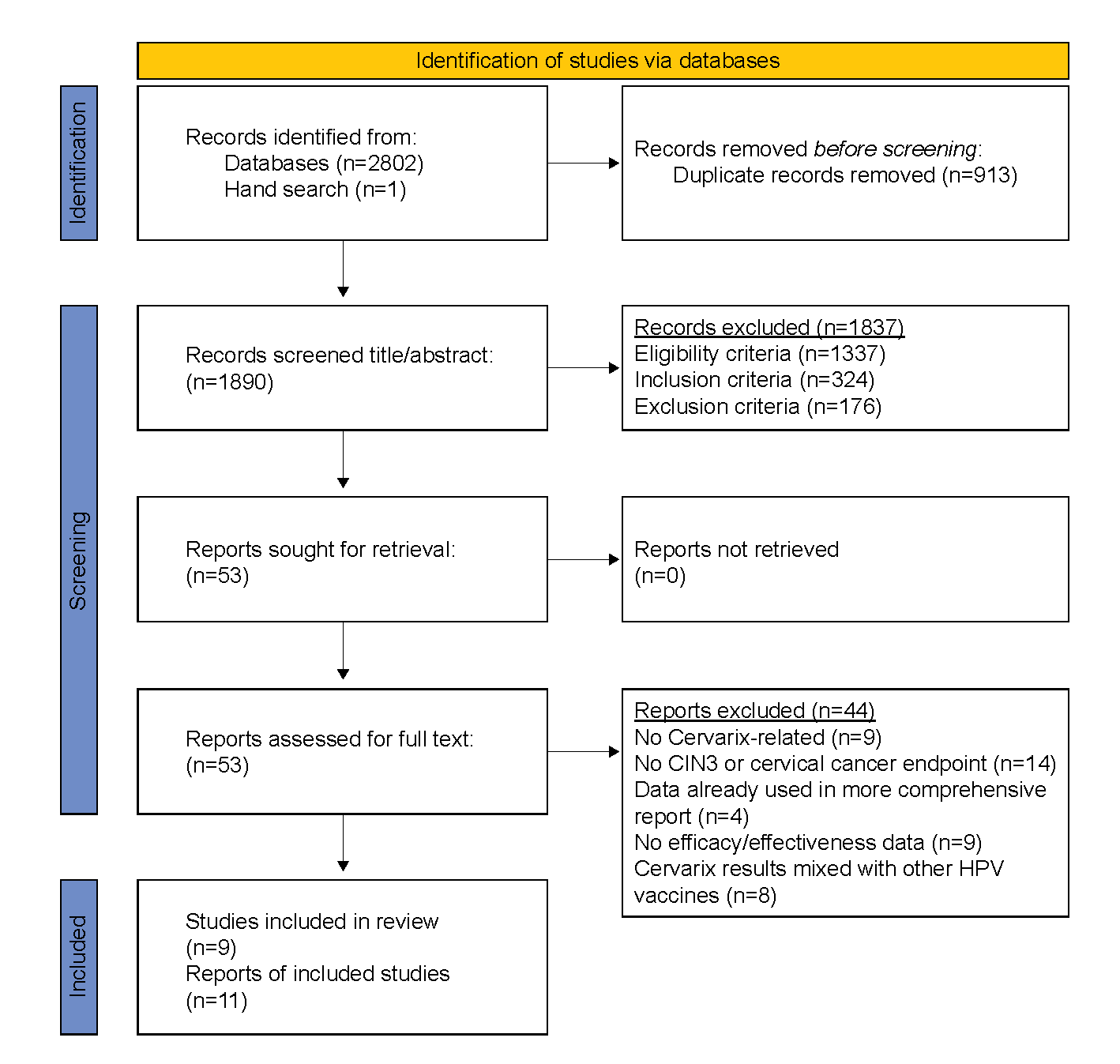
*

CIN3=cervical intraepithelial neoplasia grade 3. HPV=human papillomavirus. n=number of reports. PRISMA=Preferred Reporting Items for Systematic Reviews and Meta-Analyses. Note: Two papers (ie, Porras, Shing) included reports both on vaccine efficacy and vaccine effectiveness (observational component).^1,2^

Table SII: Vaccine effects reported on different endpoints

| Author, year | N (overall) | Age at first vaccination (years) | N (age group) | Endpoints | Vaccine effects % (95% CI) |
| --- | --- | --- | --- | --- | --- |
| Wheeler, 2012^3^ | ATP-E^a^, N=16114  vaccine arm, n=8067  control arm, n=8047  TVC^b^, N=18644  vaccine arm, n=9319  control arm, n=9325  TVC-naïve^c^, N=11644  vaccine arm, n=5824  control arm, n=5820 | 15–25 | NA | Vaccine efficacy against CIN3+ associated with a composite of 12 non-vaccine HPV types, with or without HPV-16/18 co-infection, in the ATP-E cohort | 73.8 (48.3–87.9) |
|  |  |  |  | Vaccine efficacy against CIN3+ associated with a composite of 12 non-vaccine HPV types excluding HPV-16/18 co-infection, in the ATP-E cohort | 62.1 (21.8–82.9) |
|  |  |  |  | Vaccine efficacy against CIN3+ associated with a composite of 12 non-vaccine HPV types, with or without HPV-16/18 co-infection, in the TVC-naïve | 91.4 (65.0–99.0) |
|  |  |  |  | Vaccine efficacy against CIN3+ associated with a composite of 12 non-vaccine HPV types, excluding HPV-16/18 co-infection, in the TVC-naïve | 81.9 (17.1–98.1) |
|  |  |  |  | Vaccine efficacy against CIN3+ associated with a composite of 12 non-vaccine HPV types, with or without HPV-16/18 co-infection, in the TVC | 47.5 (22.8–64.8) |
|  |  |  |  | Vaccine efficacy against CIN3+ associated with a composite of 12 non-vaccine HPV type, excluding HPV-16/18 co-infection, in the TVC | 40.0 (1.1–64.2) |
| Lehtinen, 2012^4^ | ATP-E, N=16114  vaccine arm, n=8067  control arm, n=8047  TVC, N=18644  vaccine arm, n=9319  control arm, n=9325  TVC-naïve, N=11644  vaccine arm, n=5824  control arm, n=5820 | 15–25 | NA | Vaccine efficacy against CIN3+ associated with HPV-16/18 in TVC-naïve | 100 (85.5–100) |
|  |  | 15–25 |  | Vaccine efficacy against CIN3+ associated with HPV-16/18 in TVC | 45.7 (22.9–62.2) |
|  |  | 15–25 |  | Vaccine efficacy against CIN3+ associated with HPV-16/18 in ATP-E cohort | 91.7 (66.6–99.1) |
|  |  | 15–25 |  | Vaccine efficacy against CIN3+ associated with HPV-16 in ATP-E cohort | 90.2 (59.7–98.9) |
|  |  | 15–25 |  | Vaccine efficacy against CIN3+ associated with HPV-18 in ATP-E cohort | 100 (-8.2–100) |
|  |  | 15–25 |  | Vaccine efficacy against all CIN3+ (irrespective of HPV type in the lesion and including lesions with no HPV DNA detected) in the TVC-naïve | 93.2 (78.9–98.7) |
|  |  | 15–25 |  | Vaccine efficacy against all CIN3+ (irrespective of HPV type in the lesion) in the TVC | 45.6 (28.8–58.7) |
|  |  | 15–25 |  | Vaccine efficacy against all AIS HPV 16/18-related in the TVC-naïve | 100 (15.5–100) |
|  |  | 15–25 |  | Vaccine efficacy against all AIS HPV 16/18-related in the TVC | 70.0 (-16.6–94.7) |
|  |  | 15–25 |  | Vaccine efficacy against all AIS irrespective of HPV DNA in the lesion in the TVC-naïve | 100 (31.0–100) |
|  |  | 15–25 |  | Vaccine efficacy against all AIS irrespective of HPV DNA in the lesion in the TVC | 76.9 (16.0–95.8) |
|  |  | 15–25 |  | Vaccine efficacy against AIS associated with HPV-16/18 in ATP-E cohort | 100 (-8.6–100) |
|  |  | 15–25 |  | Vaccine efficacy against AIS associated with HPV-16 in ATP-E cohort | 100 (48.4–100) |
|  |  | 15–25 |  | Vaccine efficacy against AIS associated with HPV-18 in ATP-E cohort | 100 (-3768.9–100) |
|  |  | 15–17 |  | Vaccine efficacy against CIN3+ associated with HPV-16/18 in TVC-naïve^d^ | 100 (69.4–100) |
|  |  | 18–25 |  | Vaccine efficacy against CIN3+ associated with HPV-16/18 in TVC-naïve | 100 (67.8–100) |
|  |  | 18–20 |  | Vaccine efficacy against CIN3+ associated with HPV-16/18 in TVC-naïve^d^ | 100 (39.5–100) |
|  |  | 21–25 |  | Vaccine efficacy against CIN3+ associated with HPV-16/18 in TVC-naïve^d^ | 100 (-4.6–100) |
|  |  | 15–17 |  | Vaccine efficacy against CIN3+ associated with HPV-16/18 in TVC^d^ | 80.5 (55.6–92.7) |
|  |  | 18–25 |  | Vaccine efficacy against CIN3+ associated with HPV-16/18 in TVC | 24.2 (-14.1–50.0) |
|  |  | 18–20 |  | Vaccine efficacy against CIN3+ associated with HPV-16/18 in TVC^d^ | 56.3 (13.6–79.1) |
|  |  | 21–25 |  | Vaccine efficacy against CIN3+ associated with HPV-16/18 in TVC^d^ | -10.1 (-90.5–36.1) |
|  |  | 15–17 |  | Vaccine efficacy against all CIN3+ (irrespective of HPV type in the lesion and including lesions with no HPV DNA detected) in the TVC-naïve^e^ | 91.5 (65.9–99.0) |
|  |  | 18–25 |  | Vaccine efficacy against all CIN3+ (irrespective of HPV type in the lesion and including lesions with no HPV DNA detected) in the TVC-naïve | 95.1 (69.3–99.9) |
|  |  | 18–20 |  | Vaccine efficacy against all CIN3+ (irrespective of HPV type in the lesion and including lesions with no HPV DNA detected) in the TVC-naïve^e^ | 90.6 (35.5–99.8) |
|  |  | 21–25 |  | Vaccine efficacy against all CIN3+ (irrespective of HPV type in the lesion and including lesions with no HPV DNA detected) in the TVC-naïve^e^ | 100 (51.4–100) |
|  |  | 15–17 |  | Vaccine efficacy against all CIN3+ (irrespective of HPV type in the lesion) in the TVC^e^ | 65.5 (42.5–80.0) |
|  |  | 18–25 |  | Vaccine efficacy against all CIN3+ (irrespective of HPV type in the lesion) in the TVC | 33.1 (7.5–51.9) |
|  |  | 18–20 |  | Vaccine efficacy against all CIN3+ (irrespective of HPV type in the lesion) in the TVC^e^ | 49.5 (13.9–71.2) |
|  |  | 21–25 |  | Vaccine efficacy against all CIN3+ (irrespective of HPV type in the lesion) in the TVC^e^ | 19.5 (-22.7–47.4) |

| Konno, 2014^5^ | TVC combined, N=1040  vaccine arm, n=519  control arm, n=521  TVC-naïve combined, N=565  vaccine arm, n=281  control arm, n=284 | 20–25 | NA | Vaccine efficacy against CIN3+ irrespective of the HPV type in the TVC-naive (over the combined 4-year study period of initial and follow-up studies)^e^  Vaccine efficacy against CIN3+ irrespective of the HPV type in the TVC (over the combined 4-year study period of initial and follow-up studies)^e^ | 100 (-417.0–100) |
| --- | --- | --- | --- | --- | --- |
|  |  |  |  |  | 36.4 (-57.8–75.7) |
| Lehtinen, | N=18092  vaccinated arm, n=2465  unvaccinated arm,  n=15627 | 16–17 | NA | Vaccine effectiveness against CIN3+ caused by HPV16 | 22 (-160–73) |
| 2017^6^ |  |  |  | Vaccine effectiveness against CIN3+ caused by HPV18 | 100 (-1500–100) |
|  |  |  |  | Vaccine effectiveness against CIN3+ caused by HPV16/18 | 27 (-140–74) |
|  |  |  |  | Vaccine effectiveness against CIN3+ caused by HPV16/31/33/35/52/58 | 53 (-48–83) |
|  |  |  |  | Vaccine effectiveness against CIN3+ caused by HPV31/33/35/52/58 (excluding co-infections with HPV16) | 100 (-65–100) |
|  |  |  |  | Vaccine effectiveness against CIN3+ caused by A9=HPV31/33/35/52/58 and A7=HPV39/45/59/68 (excluding co-infections with 16/18) | 100 (-55–100) |
|  |  |  |  | Vaccine effectiveness against CIN3+ caused by HPV31/33/45 | 100 (-120–100) |
|  |  |  |  | Vaccine effectiveness against CIN3+ caused by HPV6/11/16/18/31/33/45/51/74 (all protected types) | 50 (-60–82) |
|  |  |  |  | Vaccine effectiveness against CIN3+ caused by HPV6/11/31/33/45/51/74 (all protected types excluding co-infections with 16/18) | 100 (-120–100) |
|  |  |  |  | Vaccine effectiveness against CIN3+ caused by HPV34/35/39/40/42/43/44/52/53/54/56/58/59/66/68/70/73 (all non-protected types excluding co-infections with 16/18) | 100 (-480–100) |
|  |  |  |  | Vaccine effectiveness against CIN3+ caused by all detected HPV types | 56 (-38–84) |
|  |  |  |  | Vaccine effectiveness against CIN3+ caused by all detected HPV types (HPV positive and HPV negative baseline, excluding co-infections with 16/18) | 100 (-55–100) |
|  |  |  |  | Vaccine effectiveness against CIN3+ caused by Total (original FCR registered CIN3+ diagnoses) | 59 (-26–85) |
|  |  |  |  | Vaccine effectiveness against CIN3+ caused by Total All, irrespective of HPV type, this includes the re-review of histopathological block retrieval and re-analysis | 66 (8.4–88) |
| Porras, 2020^1^ | Analytical cohort (0–4 years), N=5312  vaccine arm, n=2635  control arm, n=2677  Analytical cohort  (7–11 years), N=4603  vaccinated arm, n=2073  unvaccinated arm, n=2530 | 18–25 | NA | Vaccine efficacy against CIN3+ caused by HPV 16/18 at year 4 post-vaccination (analytical cohort with original control group)^d^ | 66.4 (-175–97.3) |
|  |  |  |  | Vaccine effectiveness against CIN3+ caused by HPV 16/18 at year 7 post-vaccination (analytical cohort with unvaccinated new control group) | 100 (-40.1–100) |
|  |  |  |  | Vaccine effectiveness against CIN3+ caused by HPV 16/18 at year 9 post-vaccination (analytical cohort with unvaccinated new control group) | 100 (44.0–100) |
|  |  |  |  | Vaccine effectiveness against CIN3+ caused by HPV 16/18 at year 11 post-vaccination (analytical cohort with unvaccinated new control group) | 100 (78.8–100) |
| Shing, 2022^2^ | Analytical cohort (1–4 years), N=7003  vaccine arm, n=3491  control arm, n=3512  Analytical cohort (7–11 years), N=5418  vaccinated arm, n=2826  unvaccinated arm, n=2592 | 18–25 | NA | Vaccine efficacy against incident CIN3+ irrespective of HPV type (combined 4-year period) | 25.2 (-5.0– 46.9) |
|  |  |  |  | Vaccine efficacy against incident CIN3+ caused by HPV16 or HPV18 (combined 4-year period) | 52.9 (22.4–72.1) |
|  |  |  |  | Vaccine efficacy against incident CIN3+ caused by HPV31,33, or 45 (excluding HPV16 or HPV18 coinfection) (combined 4-year period) | -16.1 (-149.0–45.3) |
|  |  |  |  | Vaccine efficacy against incident CIN3+ caused by HPV types other than HPV16, 18, 31, 33, or 45 (combined 4-year period) | -17.4 (-123.2–37.8) |
|  |  |  |  | Vaccine effectiveness against incident CIN3+ irrespective of HPV type (combined years 7–11-year period)^e^ | 14.4 (-23.4–40.7) |
|  |  |  |  | Vaccine effectiveness against incident CIN3+ caused by HPV16 or HPV18 (combined years 7–11-year period)^d^ | 86.9 (65.3–96.1) |
|  |  |  |  | Vaccine effectiveness against incident CIN3+ caused by HPV31, 33, or 45 (excluding HPV16 or HPV18 coinfection) (combined years 7–11-year period) | 36.9 (-36.2–71.6) |
|  |  |  |  | Vaccine effectiveness against incident CIN3+ caused by HPV types other than HPV16, 18, 31, 33, or 45 (combined years 7–11-year period) | -135.0 (-329.8 to –33.5) |
|  |  |  |  | Vaccine effectiveness against incident CIN3+ irrespective of HPV type (combined 11-year period) | 19.5 (-3.3–37.5) |
|  |  |  |  | Vaccine effectiveness against incident CIN3+ caused by HPV16 or HPV18 (combined 11-year period) | 67.9 (51.1–80.4) |
|  |  |  |  | Vaccine effectiveness against incident CIN3+ caused by HPV31, 33, or 45 (excluding HPV16 or HPV18 coinfection) (combined 11-year period) | 16.6 (-40.6–52.4) |
|  |  |  |  | Vaccine effectiveness against incident CIN3+ caused by HPV types other than HPV16, 18, 31, 33, or 45 (combined 11-year period) | -81.7 (-190.6 to –19.9) |

| Palmer, 2019^7^ | N=138692  Zero doses (unvaccinated) n=64026  One dose, n=2051  Two doses, n=4135  Three doses, n=68480 | 12–13 | N=16200 | Vaccine effectiveness against CIN3+^e, f, g^ | 86 (75–92) |
| --- | --- | --- | --- | --- | --- |
|  |  | 14 | N=5409 | Vaccine effectiveness against CIN3+^e^ | 82 (57–93) |
|  |  | 15 | N=16532 | Vaccine effectiveness against CIN3+^e^ | 71 (56–81) |
|  |  | 16 | N=17511 | Vaccine effectiveness against CIN3+^e^ | 73 (59–82) |
|  |  | 17 | N=8711 | Vaccine effectiveness against CIN3+^e^ | 45 (17–64) |
|  |  | ≥18 | N=4117 | Vaccine effectiveness against CIN3+^e^ | 15 (-37–48) |
|  |  | ≤17 | N=15678 | Vaccine effectiveness against CIN3+, born ≥1991 (unvaccinated) | 18 (-7–37) |
|  |  | 12–13 | N=48348 | Vaccine effectiveness against CIN3, born 1995–1996 (unvaccinated) | 100 (69–100) |
| Falcaro, 2021^8^ | 13.7 million years of follow-up | 12–13 | NA | Vaccine effectiveness against CIN3^h^ | 97 (96–98) |
|  |  | 14–16 |  | Vaccine effectiveness against CIN3 | 75 (72–77) |
|  |  | 16–18 |  | Vaccine effectiveness against CIN3 | 39 (36–41) |
|  |  | 12–13 |  | Vaccine effectiveness against cervical cancer | 87 (72–94) |
|  |  | 14–16 |  | Vaccine effectiveness against cervical cancer | 62 (52–71) |
|  |  | 16–18 |  | Vaccine effectiveness against cervical cancer | 34 (25–41) |
| Rebolj, 2022^9^ | N=108138  vaccinated, n=64274  unvaccinated, n=43863 | 14–17 | NA | Vaccine effectiveness against HR-HPV positive CIN3+ (HR-HPV+/cytology+ primary screening test)^e,^ ^i^ | 79 (73–83) |
|  |  |  |  | Vaccine effectiveness against HPV 16/18-related CIN3+^d^ | 87 (80–91) |
|  |  |  |  | Vaccine effectiveness against CIN3+ by ‘Other’ HPV-related (excludes co-infections with HPV16/18)^j^ | 57 (25–75) |
|  |  |  |  | Vaccine effectiveness against cervical cancer | 64 (-91–93) |
|  |  |  |  |  |  |

AIS=adenocarcinoma in situ. ATP-E=according-to-protocol for efficacy cohort. CI=confidence interval. CIN3+=grade 3 cervical intraepithelial neoplasia or worse. FCR=Finnish Cancer Registry. HPV=human papillomavirus. HR-HPV=high-risk human papillomavirus. N=total (overall). n=number of participants in each arm. NA=not applicable. TVC=total vaccinated cohort. VE=vaccine effects.

^a^Participants received three doses of vaccine and were HPV DNA negative at baseline. ^b^Participants received at least one dose of vaccine, irrespective of baseline HPV DNA status. ^c^Participants received at least one dose of vaccine and were HPV DNA negative at baseline. ^d^This subcohort was included in the analysis 1. ^e^This subcohort was included in the analysis 2. ^f^Vaccine effectiveness calculated as VE=(1-odds ratio)*100. ^g^Results for three doses of vaccine. ^h^Vaccine effectiveness calculated as VE=(1-IRR)*100. (Adjusted IRR model 3). ^i^14 HR-HPV types: 16, 18, 31, 33, 35, 39, 45, 51, 52, 56, 58, 59, 66, 68. ^j^‘Other’ 12 HR-HPV types: 31, 33, 35, 39, 45, 51, 52, 56, 58, 59, 66, 68.

For this study, vaccine efficacy refers to outcomes/results recorded in controlled conditions (RCTs), vaccine effectiveness refers to outcomes/results recorded in real-world conditions (observational studies), and VE is a combination of vaccine efficacy and vaccine effectiveness.

Table SIII: Final outcomes and endpoints for the meta-regression analyses

| Author, year | Endpoint | HPV type | N of doses | Age at first vaccination | Time since vaccination (years) |
| --- | --- | --- | --- | --- | --- |
| Analysis 1_CIN3+, HPV16/18 RCT/observational combined | | | | | |
| RCT, vaccine efficacy | | | | | |
| Lehtinen, 2012^4^ | CIN3+ | HPV16/18 | At least one dose (TVC and TVC-naïve) | 15–17 years  18–20 years  21–25 years | 0–4 |
| Porras, 2020^1^ | CIN3+ | HPV16/18 | At least one dose (TVC-naïve) | 18–25 years | 0–4 |
| Observational; population-based surveillance, vaccine effectiveness | | | | | |
| Shing, 2022^2^ | CIN3+ | HPV16/18 | At least one dose (TVC) | 18–25 years | 7–11 |
| Rebolj, 2022^9^ | CIN3+ | HPV16/18 | Three doses | 14–17 years | 7–11 |
| Analysis 2_CIN3+, irrespective of HPV type RCT/observational combined | | | | | |
| RCT, vaccine efficacy | | | | | |
| Konno, 2014^5^ | CIN3+ | Irrespective of HPV type | At least one dose (TVC and TVC-naïve) | 20–25 years | 0–4 |
| Lehtinen, 2012^4^ | CIN3+ | Irrespective of HPV type | At least one dose (TVC and TVC-naïve) | 15–17 years  18–20 years  21–25 years | 0–4 |
| Observational; population-based surveillance, vaccine effectiveness | | | | | |
| Palmer, 2019^7^ | CIN3+ | Histological diagnosis (no HPV testing results). Considered as ‘irrespective of HPV type’ | Three doses | 12–13 years  14 years  15 years  16 years  17 years  ≥18 years | 0–8  0–6  0–5  0–4  0–3  0–2 |
| Shing, 2022^2^ | CIN3+ | Irrespective of HPV type | At least one dose | 18–25 years | 7–11 |
| Rebolj, 2022^9^ | CIN3+ | HR-HPV (16, 18, 31, 33, 35, 39, 45, 51, 52, 56, 58, 59, 66, 68). Considered as ‘irrespective of HPV type’ | Three doses | 14–17 years | 7–11 |
| Analysis 3_CIN3+, HPV16/18, RCT | | | | | |
| RCT, vaccine efficacy | | | | | |
| Lehtinen, 2012^4^ | CIN3+ | HPV16/18 | At least one dose (TVC and TVC-naïve) | 15–17 years  18–20 years  21–25 years | 0–4 |
| Porras, 2020^1^ | CIN3+ | HPV16/18 | At least one dose (TVC-naïve) | 18–25 years | 0–4 |
| Shing, 2022^2^ | CIN3+ | HPV16/18 | At least one dose (TVC) | 18–25 years | 0–4 |
| Analysis 4_CIN3+, HPV16/18, observational, vaccine effectiveness | | | | | |
| Lehtinen, 2017^6^ | CIN3+ | HPV16/18 | At least one dose | 16–17 years | 0–10 |
| Shing, 2022^2^ | CIN3+ | HPV16/18 | At least one dose | 18–25 years | 7–11 |
| Rebolj, 2022^9^ | CIN3+ | HPV16/18 | Three doses | 14–17 years | 7–11 |
| Analysis 5_CIN3+, irrespective of HPV type, RCT, vaccine efficacy | | | | | |
| Konno, 2014^5^ | CIN3+ | Irrespective of HPV type | At least one dose (TVC and TVC-naïve) | 20–25 years | 0–4 |
| Lehtinen, 2012^4^ | CIN3+ | Irrespective of HPV type | At least one dose (TVC and TVC-naïve) | 15–17 years  18–20 years  21–25 years | 0–4 |
| Shing, 2022^2^ | CIN3+ | Irrespective of HPV type | At least one dose (TVC) | 18–25 years | 1–4 |
| Analysis 6_CIN3+, irrespective of HPV type, observational, vaccine effectiveness | | | | | |
| Lehtinen, 2017^6^ | CIN3+ | Irrespective of HPV type | At least one dose | 16–17 years | 0–10 |
| Palmer, 2019^7^ | CIN3+ | Histological diagnosis (no HPV testing results). Considered as ‘irrespective of HPV type’ | Three doses | 12–13 years  14 years  15 years  16 years  17 years  ≥18 years | 0–8  0–6  0–5  0–4  0–3  0–2 |
| Shing, 2022^2^ | CIN3+ | Irrespective of HPV type | At least one dose | 18–25 years | 7–11 |
| Rebolj, 2022^9^ | CIN3+ | HR-HPV (16, 18, 31, 33, 35, 39, 45, 51, 52, 56, 58, 59, 66, 68). Considered as ‘irrespective of HPV type’ | Three doses | 14–17 years | 7–11 |

CIN3+=grade 3 cervical intraepithelial neoplasia or worse. HPV=human papillomavirus. HR-HPV=high-risk human papillomavirus. N=total (overall). RCT=randomised controlled trial. TVC=total vaccinated cohort. VE= vaccine effects.

For this study, vaccine efficacy refers to outcomes/results recorded in controlled conditions (RCTs), vaccine effectiveness refers to outcomes/results recorded in real-world conditions (observational studies), and VE is a combination of vaccine efficacy and vaccine effectiveness.

**Quality control and quality assurance**

**Quality assessment of RCTs**

Follow-up post-hoc studies of RCTs by Lehtinen (2012),^4^ Porras (2020)^1^ and Shing (2022)^2^ presented low risk of bias (**Table SIV** and **Figure S2**). The main feature of these studies is that double-blinding was kept beyond the 3-year RCT duration up to the end of the 4-year follow-up (48 months). Therefore, participants, study personnel and investigators were blinded to the intervention allocation. In contrast, this was not the case for Konno (2014),^5^ where the blinding was broken at the end of the primary RCT at 36 months. Therefore, participants and carers were aware of the intervention allocation during the follow-up period. This is why this study had the overall judgement of presenting ‘some concerns’. However, laboratory staff were blinded to the intervention which prevents bias at the diagnosis and assessment of the outcome and, ultimately, the follow-up unblinding was expected to not have influenced the efficacy assessment. Another important aspect is that the Konno (2014) study was not powered to evaluate vaccine efficacy against CIN3+. Hence the large 95% CIs for the results on this outcome (**Table SII**). This aspect was addressed in the analysis phase (meta-regression analysis).

Overall, completeness of all follow-up studies was quite high, and losses were not selective, leaving both arms balanced at completion of the study.

**Table SIV: Risk of bias assessment of RCTs from the systematic review^10^**

^
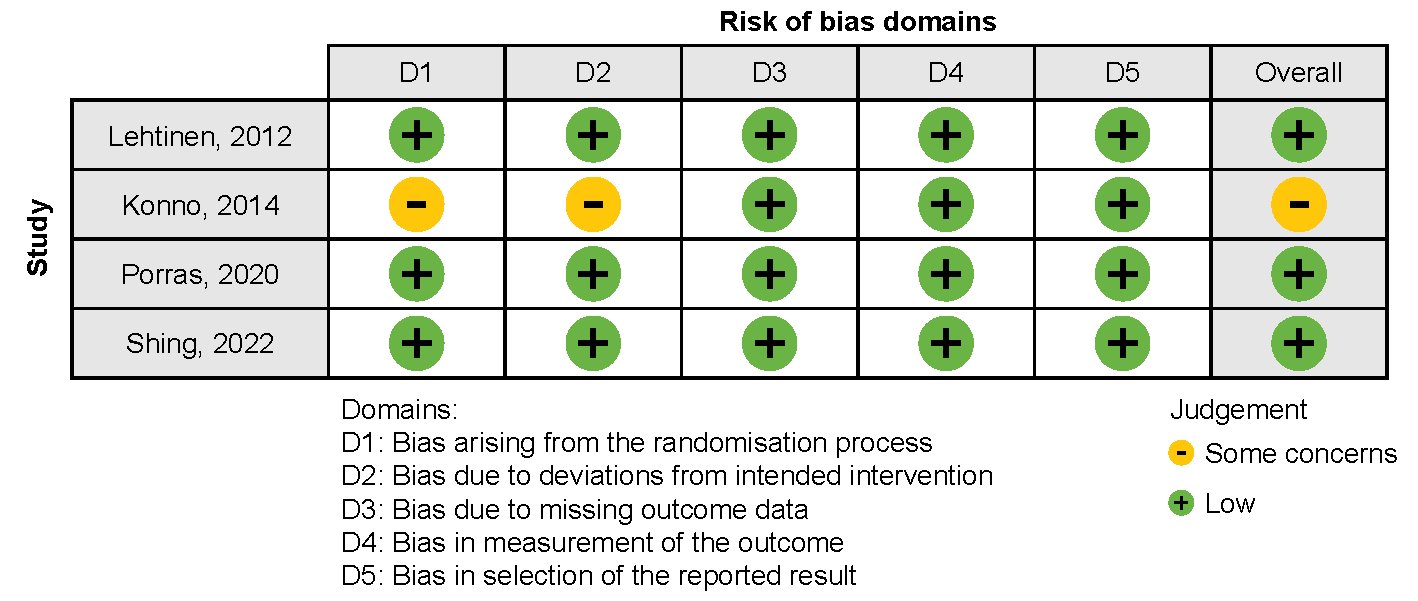
^

## **Fig. S2: Summary of quality assessment rating regarding bias (RCTs)^10^**


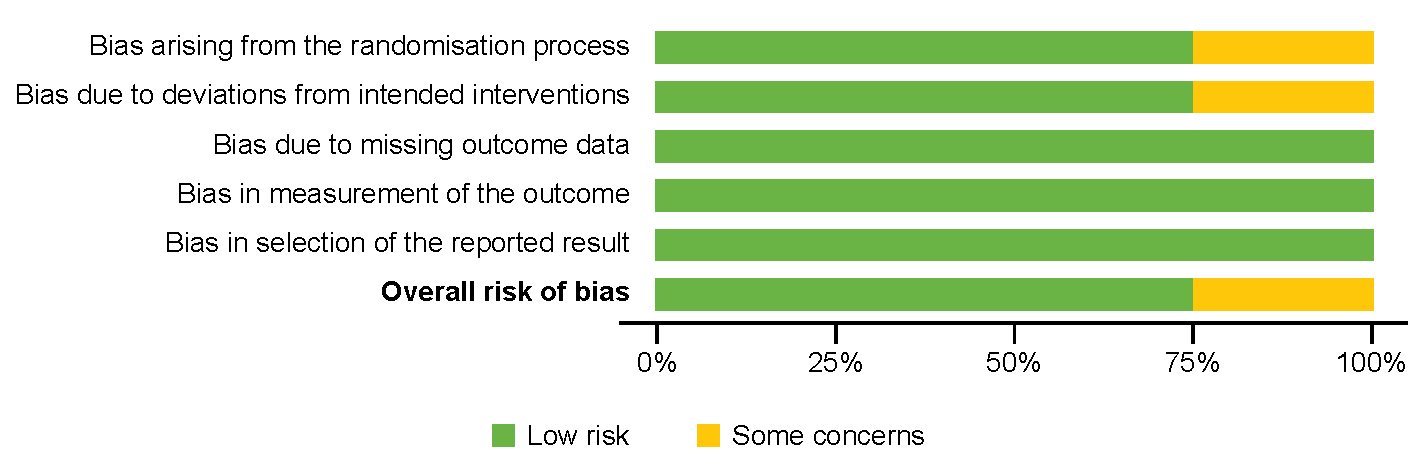


D=domain. RCT=randomised clinical trial.

The 0–100% scale represents the global risk of bias attributable to every bias domain and overall, for all included RCTs.

**Quality assessment of observational studies**

All the included studies were considered to have at least moderate risk of bias, and two of the five studies included were at high (serious) risk of bias including Palmer (2019)^7^ and Rebolj (2022)^9^ (**Table SV** and **Figure S3**). These two studies had one or two domains at high risk (mainly confounding and information of outcome). The uptake of screening in fully vaccinated women aged 20 or 21 years was 51% and only 23% in unvaccinated women, and this may have overestimated vaccine effectiveness.^7^ On the other hand, authors adjusted by immunisation status and age at which the first dose was administered, and by year of birth in unvaccinated women, respectively. The analysis also adjusted for socioeconomic status (deprivation and rurality scores). In Rebolj (2022), individual vaccination status was unknown. The age and calendar year specific probability that a woman was vaccinated was estimated from the official national statistics for vaccination with three doses in the general population, available by school cohort. However, these two studies were population-based retrospective cohort studies limiting the risk of selection bias. Nevertheless, the overall judgement was that both studies addressed bias and confounding in an appropriate manner in the analytical phase considering the limitations of the retrospective population-based registry linked study design.^7,9^

An important source of confounding in observational studies is related to human papilloma virus (HPV) acquisition. The population-based studies did not determine HPV baseline status to assess for prevalent infection at the time of vaccination as pre-vaccination cervical screening is not standard of care. To address this, many studies allowed for buffer time between the vaccination and outcome assessment (cervical screening). Another relevant source of confounding in observational studies determining HPV vaccine effectiveness is differences in risk of HPV acquisition between vaccinated and unvaccinated participants. In those studies, other than stemming from national surveillance, baseline characteristics of the participants were assessed, most importantly in relation to sexual behaviour and activity, and adjusted for (i.e., Porras^1^ and Shing^2^); in other instances, sexual debut age was very similar between the vaccinated and unvaccinated arms.^6^

**
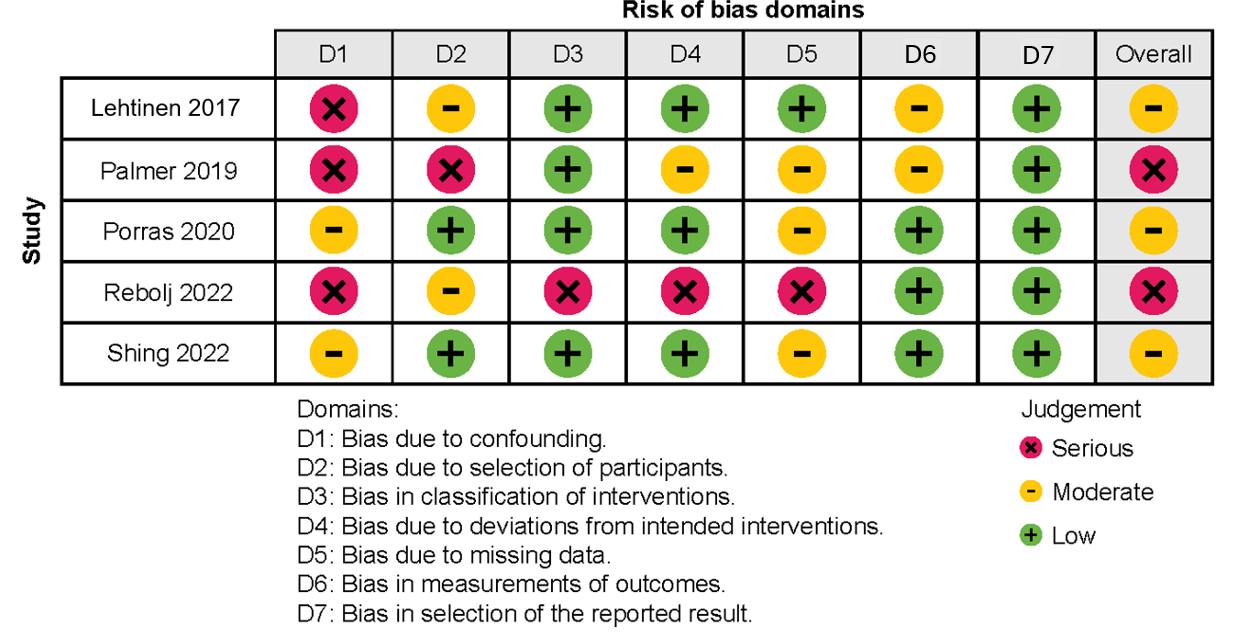
Table SV: Risk of bias of observational studies from the systematic review^10^**

D7

D6

**Fig. S3: Summary of quality assessment rating regarding bias (observational studies)^10^**


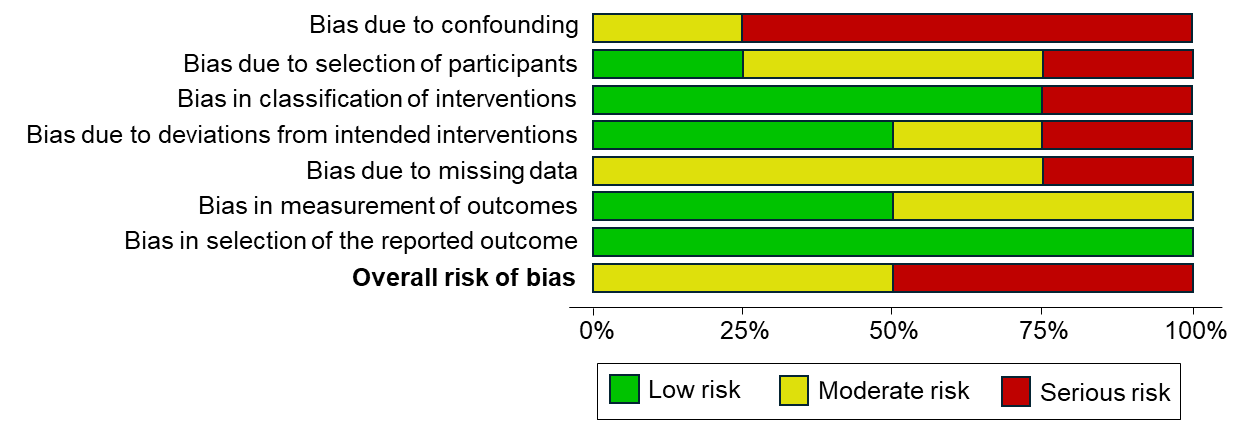


Note: The 0–100% scale represents the global risk of bias attributable to every bias domain and overall, for all selected observational studies.

**
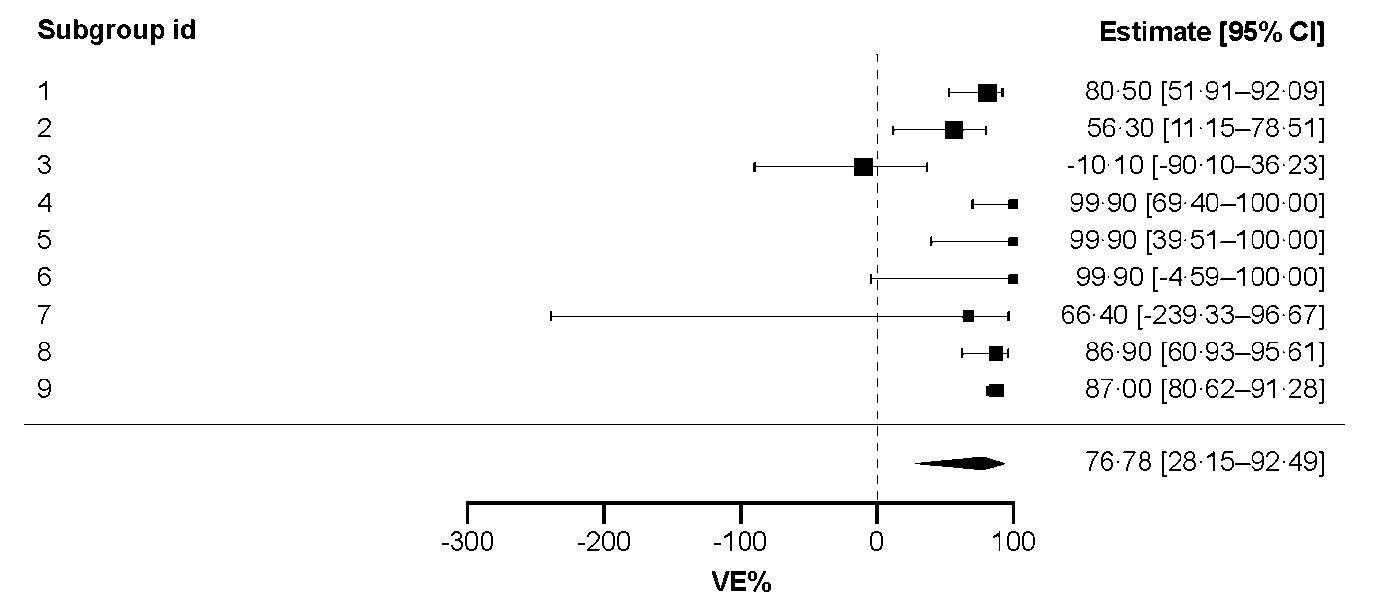
Fig. S4: Pooled estimated vaccine effects of HPV16/18-AS04 on CIN3+ caused by HPV16/18 types**

CI=confidence interval. CIN3+= grade 3 cervical intraepithelial neoplasia or worse. HPV=human papillomavirus. VE=vaccine effects. TVC=total vaccinated cohort.

Note for interpretation of graphs:

**Subgroup**

1=Lehtinen 2012,^4^ age at first vaccination 15–17 years, TVC, time since vaccination 0–4 years.

2=Lehtinen 2012,^4^ age at first vaccination 18–20 years, TVC, time since vaccination 0–4 years.

3=Lehtinen 2012,^4^ age at first vaccination 21–25 years, TVC, time since vaccination 0–4 years.

4=Lehtinen 2012,^4^ age at first vaccination 15–17 years, TVC-naïve, time since vaccination 0–4 years.

5=Lehtinen 2012,^4^ age at first vaccination 18–20 years, TVC-naïve, time since vaccination 0–4 years.

6=Lehtinen 2012,^4^ age at first vaccination 21–25 years, TVC-naïve, time since vaccination 0–4 years.

7=Porras 2020,^1^ age at first vaccination 18–25 years, TVC-naïve, time since vaccination 0–4 years.

8=Shing 2022,^2^ age at first vaccination 18–25 years, TVC, time since vaccination 7–11 years.

9=Rebolj 2022,^9^ age at first vaccination 14–17 years, TVC, time since vaccination 7–11 years.

*σ^2^*=0.62; *I^2^*=75%.

**Table SVI: Interpretation of results of the risk of bias assessment for observational studies**

| Low |  | The study is comparable to a well-performed randomised trial regarding this domain |
| --- | --- | --- |
| Moderate |  | The study is sound for a non-randomised study regarding this domain but cannot be considered comparable to a well-performed randomised trial |
| Serious |  | The study has some important problems |

Table SVII: Multiparametric meta-regression model parameters (analysis 1)

|  |  | Estimate | Standard errors | t value | Degrees of freedom | p value | 95% CI |
| --- | --- | --- | --- | --- | --- | --- | --- |
| **Variables** | **TVC** | 1.69 | 1.04 | 1.62 | 6 | 0.16 | -0.86–4.23 |
|  | **Age group** | 0.23 | 0.06 | 3.78 | 6 | 0.01 | 0.08–0.39 |
|  | **Intercept** | -7.44 | 1.60 | -4.66 | 6 | 0.004 | -11.34 to -3.54 |

*σ^2^*=0.23; *I^2^*=46%; pseudo *R^2^*=62%.

CI=confidence interval. TVC=total vaccinated cohort.

Funding was declared in all selected papers. The PATRICIA trial^4^ and the Japanese trial^5^ were funded and coordinated by GlaxoSmithKline Biologicals. The Finnish observational study^6^ received funding from GlaxoSmithKline Biologicals and the Academy of Finland. The Costa Rica Vaccine Trial follow-up studies^1,2^ were funded by the US National Cancer Institute with funding support from the National Institutes of Health Office of Research on Women’s Health. GSK contributed vaccines to this trial. The Scottish study^7^ was part of the routine work of Health Protection Scotland (Scottish National Health Service). The British studies^8,9^ were carried out by Public Health England in collaboration with Cancer Research UK.

**Results of univariate analysis of main analysis (analysis 1)**

**Figure S5: Univariate effect of analytical cohort on vaccine efficacy/effectiveness on CIN3+ caused by HPV vaccine types**


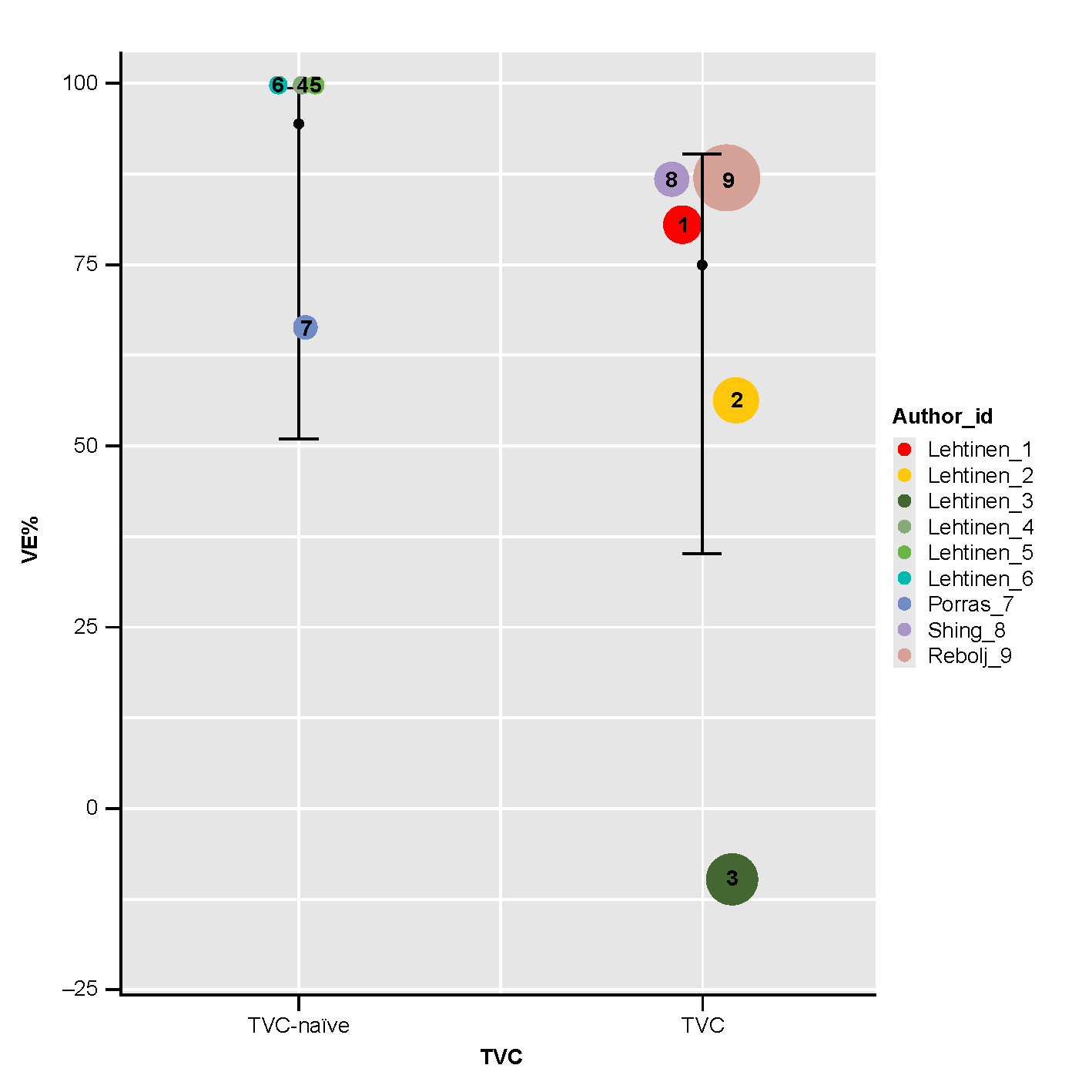


CI=confidence interval. CIN3+=grade 3 cervical intraepithelial neoplasia or worse. HPV= human papillomavirus. id=identity. TVC=total vaccinated cohort. VE=vaccine effect.

Note for interpretation of graphs:

Subgroup

1=Lehtinen 2012,^4^ age at first vaccination 15–17 years, TVC, time since vaccination 0–4 years.

2=Lehtinen 2012,^4^ age at first vaccination 18–20 years, TVC, time since vaccination 0–4 years.

3=Lehtinen 2012,^4^ age at first vaccination 21–25 years, TVC, time since vaccination 0–4 years.

4=Lehtinen 2012,^4^ age at first vaccination 15–17 years, TVC-naïve, time since vaccination 0–4 years.

5=Lehtinen 2012,^4^ age at first vaccination 18–20 years, TVC-naïve, time since vaccination 0–4 years.

6=Lehtinen 2012,^4^ age at first vaccination 21–25 years, TVC-naïve, time since vaccination 0–4 years.

7=Porras 2020,^1^ age at first vaccination 18–25 years, TVC-naïve, time since vaccination 0–4 years.

8=Shing 2022,^2^ age at first vaccination 18–25 years, TVC, time since vaccination 7–11 years.

9=Rebolj 2022,^9^ age at first vaccination 14–17 years, TVC, time since vaccination 7–11 years.

The figure above shows the observed and the predicted VE (black line with 95% CI) as a function of the univariate covariates. Size of the bubbles is proportional to the inverse of the variance (corresponds to the weight in classical meta-analysis). The numbers on the bubbles represent each subgroup, with details about the age at first vaccination or the age range at the time of vaccination provided in the figure footnote.

**Figure S6: Univariate effect of study design on vaccine efficacy/effectiveness on CIN3+ caused by HPV vaccine types**


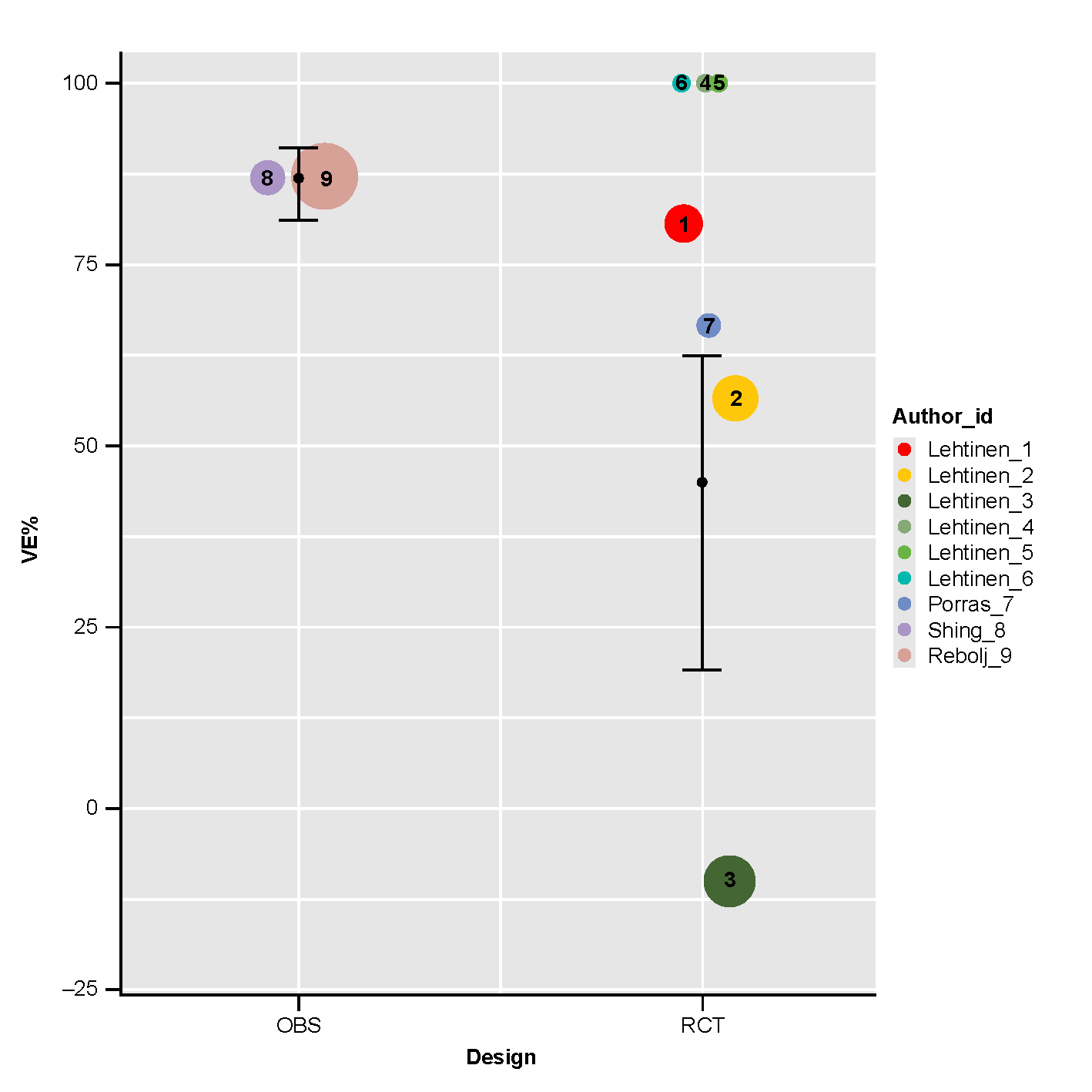


CI=confidence interval. CIN3+=grade 3 cervical intraepithelial neoplasia or worse. HPV=human papillomavirus. id=identity. OBS=observational studies. RCT=randomised control trials. TVC=total vaccinated cohort. VE=vaccine effect.

Note for interpretation of graphs:

Subgroup

1=Lehtinen 2012,^4^ age at first vaccination 15–17 years, TVC, time since vaccination 0–4 years.

2=Lehtinen 2012,^4^ age at first vaccination 18–20 years, TVC, time since vaccination 0–4 years.

3=Lehtinen 2012,^4^ age at first vaccination 21–25 years, TVC, time since vaccination 0–4 years.

4=Lehtinen 2012,^4^ age at first vaccination 15–17 years, TVC-naïve, time since vaccination 0–4 years.

5=Lehtinen 2012,^4^ age at first vaccination 18–20 years, TVC-naïve, time since vaccination 0–4 years.

6=Lehtinen 2012,^4^ age at first vaccination 21–25 years, TVC-naïve, time since vaccination 0–4 years.

7=Porras 2020,^1^ age at first vaccination 18–25 years, TVC-naïve, time since vaccination 0–4 years.

8=Shing 2022,^2^ age at first vaccination 18–25 years, TVC, time since vaccination 7–11 years.

9=Rebolj 2022,^9^ age at first vaccination 14–17 years, TVC, time since vaccination 7–11 years.

The figure above shows the observed and the predicted VE (black line with 95% CI) as a function of the univariate covariates. Size of the bubbles is proportional to the inverse of the variance (corresponds to the weight in classical meta-analysis). The numbers on the bubbles represent each subgroup, with details about the age at first vaccination or the age range at the time of vaccination provided in the figure footnote.


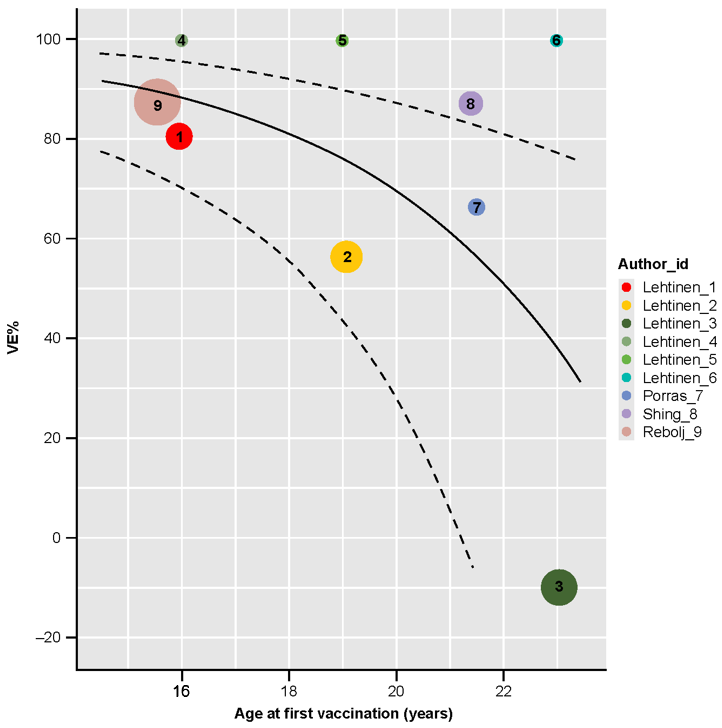
**Figure S7: Univariate effect of age at first vaccination on vaccine efficacy/effectiveness on CIN3+ caused by HPV vaccine types**

CI=confidence interval. CIN3+=grade 3 cervical intraepithelial neoplasia or worse. HPV= human papillomavirus. id=identity. TVC=total vaccinated cohort. VE=vaccine effect.

Note for interpretation of graphs:

Subgroup

1=Lehtinen 2012,^4^ age at first vaccination 15–17 years, TVC, time since vaccination 0–4 years.

2=Lehtinen 2012,^4^ age at first vaccination 18–20 years, TVC, time since vaccination 0–4 years.

3=Lehtinen 2012,^4^ age at first vaccination 21–25 years, TVC, time since vaccination 0–4 years.

4=Lehtinen 2012,^4^ age at first vaccination 15–17 years, TVC-naïve, time since vaccination 0–4 years.

5=Lehtinen 2012,^4^ age at first vaccination 18–20 years, TVC-naïve, time since vaccination 0–4 years.

6=Lehtinen 2012,^4^ age at first vaccination 21–25 years, TVC-naïve, time since vaccination 0–4 years.

7=Porras 2020,^1^ age at first vaccination 18–25 years, TVC-naïve, time since vaccination 0–4 years.

8=Shing 2022,^2^ age at first vaccination 18–25 years, TVC, time since vaccination 7–11 years.

9=Rebolj 2022,^9^ age at first vaccination 14–17 years, TVC, time since vaccination 7–11 years.

The figure above shows the observed and the predicted VE (black line with 95% CI) as a function of the univariate covariates. Size of the bubbles is proportional to the inverse of the variance (corresponds to the weight in classical meta-analysis). The numbers on the bubbles represent each subgroup, with details about the age at first vaccination or the age range at the time of vaccination provided in the figure footnote.

**Figure S8: Univariate effect of time since vaccination (time of follow-up) on vaccine efficacy/effectiveness on CIN3+ caused by HPV vaccine types
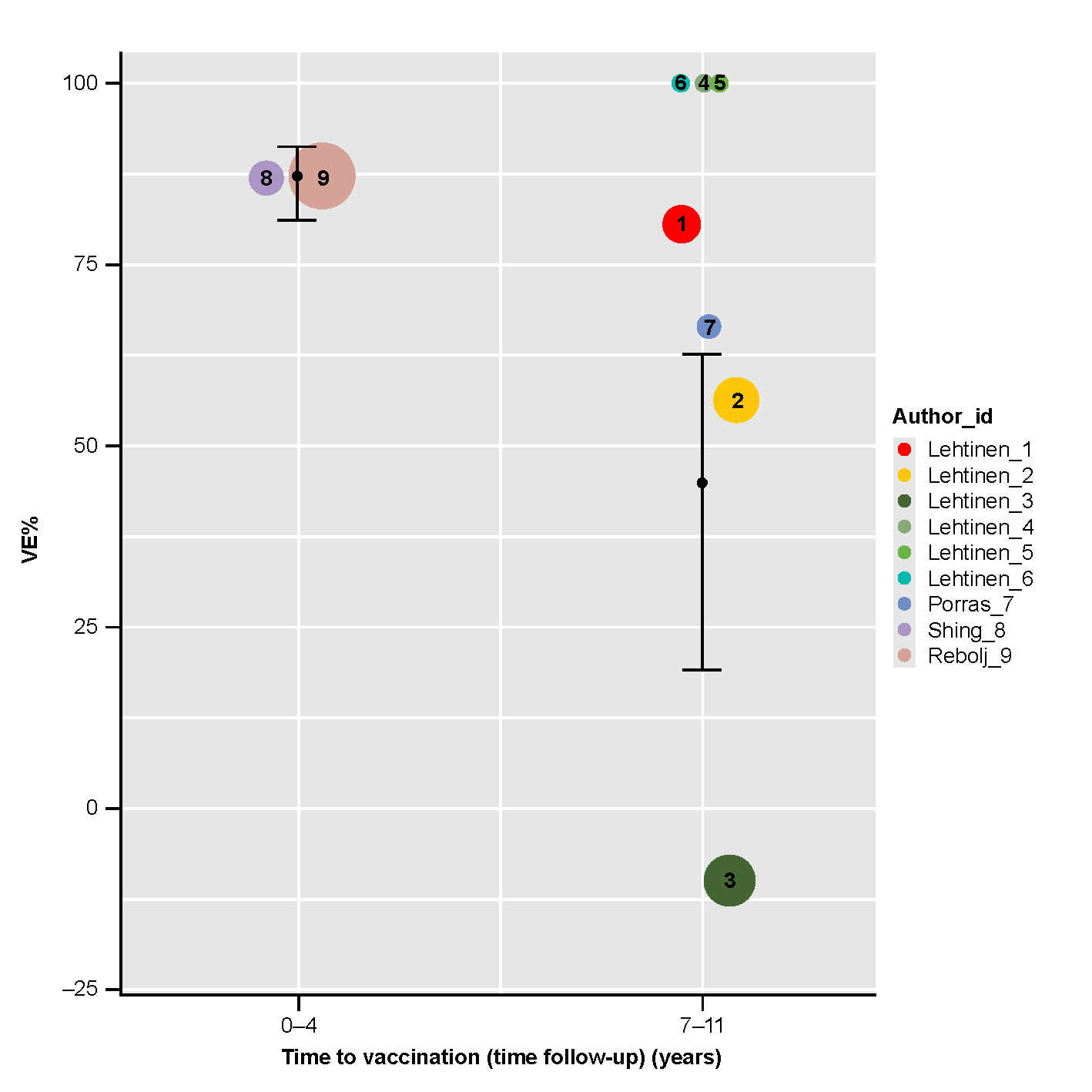
**

CI=confidence interval. CIN3+=grade 3 cervical intraepithelial neoplasia or worse. HPV= human papillomavirus. id=identity. TVC=total vaccinated cohort. VE=vaccine effect.

Note for interpretation of graphs:

Subgroup

1=Lehtinen 2012,^4^ age at first vaccination 15–17 years, TVC, time since vaccination 0–4 years.

2=Lehtinen 2012,^4^ age at first vaccination 18–20 years, TVC, time since vaccination 0–4 years.

3=Lehtinen 2012,^4^ age at first vaccination 21–25 years, TVC, time since vaccination 0–4 years.

4=Lehtinen 2012,^4^ age at first vaccination 15–17 years, TVC-naïve, time since vaccination 0–4 years.

5=Lehtinen 2012,^4^ age at first vaccination 18–20 years, TVC-naïve, time since vaccination 0–4 years.

6=Lehtinen 2012,^4^ age at first vaccination 21–25 years, TVC-naïve, time since vaccination 0–4 years.

7=Porras 2020,^1^ age at first vaccination 18–25 years, TVC-naïve, time since vaccination 0–4 years.

8=Shing 2022,^2^ age at first vaccination 18–25 years, TVC, time since vaccination 7–11 years.

9=Rebolj 2022,^9^ age at first vaccination 14–17 years, TVC, time since vaccination 7–11 years.

The figure above shows the observed and the predicted VE (black line with 95% CI) as a function of the univariate covariates. Size of the bubbles is proportional to the inverse of the variance (corresponds to the weight in classical meta-analysis). The numbers on the bubbles represent each subgroup, with details about the age at first vaccination or the age range at the time of vaccination provided in the figure footnote.

**Results of secondary analyses 2–6**

Analyses for the different scenarios were conducted following the methodology described in the main text (meta-analysis, univariate, and multiparametric meta-regression analyses). We include here results from the meta-regression that addressed the following research questions:

| **Analysis 2** | What is the combined overall efficacy/effectiveness of HPV16/18-AS04 against CIN3+ irrespective of HPV type? (RCTs and observational studies combined) |
| --- | --- |
| **Analysis 3** | What is the efficacy of HPV16/18-AS04 on CIN3+ caused by vaccine HPV types? (RCTs only) |
| **Analysis 4** | What is the effectiveness of HPV16/18-AS04 on CIN3+ caused by HPV types (observational studies only) |
| **Analysis 5** | What is the efficacy of HPV16/18-AS04 on CIN3+ caused by any HPV type? (RCTs only) |
| **Analysis 6** | What is the effectiveness of HPV16/18-AS04 on CIN3+ caused by any HPV type? (observational studies only) |

**Analysis 2: What is the combined overall efficacy/effectiveness of HPV16/18-AS04 vaccine (Cervarix^®^) on CIN3+ caused by any HPV type? (RCTs and observational studies combined).**

**Multiparametric meta-regression**

All possible combinations of predictors were evaluated and compared using Akaike information criterion (AIC; data-driven approach) to find the best model, and which predictors were the most important ones. This data-driven exploration conducted to a final model that includes the “age at first vaccination” and “analytical cohort” variables. After adjusting for the analytical cohort (TVC *vs* TVC-naïve), “age at first vaccination” resulted as the most impactful variable on the outcome (p<0.001). The heterogeneity explained by the selected model was *R^2^*=87.47%.

**Figure 2** (main text) shows the predictions (with 95% CIs, dotted line) from this data-driven selected model adjusting for “age at first vaccination” and “analytical cohort”.

**Analysis 3: What is the efficacy of HPV16/18-AS04 vaccine (Cervarix^®^) on CIN3+ caused by vaccine HPV types? (RCTs only)**

**Multiparametric meta-regression.**

All possible combinations of predictors were evaluated and compared using AIC (data-driven approach) to find the best model and which predictors were the most important ones. This data-driven exploration was conducted to a final model that includes the “age at first vaccination” and “analytical cohort” variables. After adjusting for the analytical cohort (TVC *vs* TVC-naïve), “age at first vaccination” resulted as the most impactful variable on the outcome (p=0.02). The heterogeneity explained by the selected model was *R^2^*=92.95%.

The **Figure S9** shows the predictions (with 95% CIs, dotted line) from this data-driven selected model adjusting for “age at first vaccination” and “analytical cohort”. Red and green curves represent the predicted vaccine efficacy as a function of age for TVC and TVC-naïve populations. Red and blue bubbles represent the observed vaccine efficacy of the studies with TVC and TVC-naïve population, respectively. Observed values seem to be relatively well approximated by the multiparametric model.

**Figure S9: Results of the data-driven multiparametric meta-regression analysis model (analysis 3)**


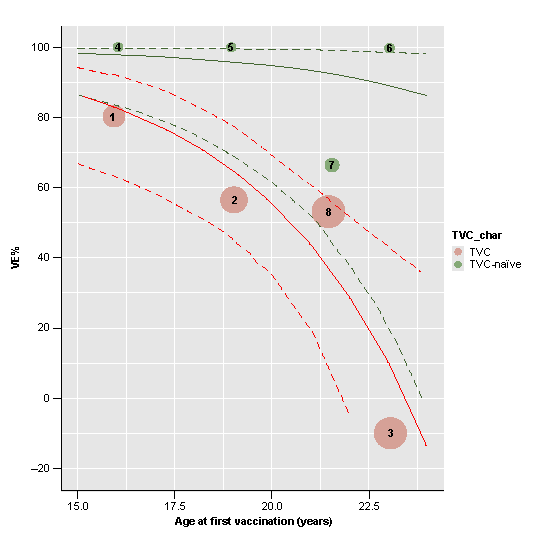


Char=characteristic. VE=vaccine effect. TVC=total vaccinated cohort.

Note for interpretation of graphs:

Subgroup

1=Lehtinen 2012,^4^ age at first vaccination 15–17 years, TVC, time since vaccination 0–4 years.

2=Lehtinen 2012,^4^ age at first vaccination 18–20 years, TVC, time since vaccination 0–4 years.

3=Lehtinen 2012,^4^ age at first vaccination 21–25 years, TVC, time since vaccination 0–4 years.

4=Lehtinen 2012,^4^ age at first vaccination 15–17 years, TVC-naïve, time since vaccination 0–4 years.

5=Lehtinen 2012,^4^ age at first vaccination 18–20 years, TVC-naïve, time since vaccination 0–4 years.

6=Lehtinen 2012,^4^ age at first vaccination 21–25 years, TVC-naïve, time since vaccination 0–4 years.

7=Porras 2020,^1^ age at first vaccination 18–25 years, TVC-naïve, time since vaccination 0–4 years.

8=Shing 2022,^2^ age at first vaccination 18–25 years, TVC, time since vaccination 1–4 years.

Size of the bubbles is proportional to the inverse of the variance (corresponds to the weight in classical meta-analysis). The numbers on the bubbles represent each subgroup, with details about the age at first vaccination or the age range at the time of vaccination provided in the figure footnote.

**Analysis 4: What is the effectiveness of HPV16/18-AS04 vaccine (Cervarix^®^) on CIN3+ caused by vaccine HPV types? (Observational studies only)**

**Multiparametric meta-regression.**

All possible combinations of predictors were evaluated and compared using AIC (data-driven approach) to find the best model, and which predictors were the most important ones. The analysis revealed that the model including “age at first vaccination” and “time since vaccination” showed a strong correlation between the two covariates. When these two covariates are included in the model, it becomes unstable, and the variance of the random effect makes it uninterpretable. Adjusting for covariates for this specific question is not meaningful.

The heterogeneity explained by the selected model was *R^2^*=100%. However, this result should be interpreted cautiously as the model was unable to properly estimate the random effect, likely because of the small number of studies included in the analysis with respect to the two covariates considered.

The following figure shows the predictions (with 95% CIs, dotted line) from this data-driven selected model adjusting for “age at first vaccination” and “time since vaccination”. Red and green curves represent the predicted vaccine effectiveness as a function of age for the time since vaccination. Red and green bubbles represent the observed vaccine effectiveness of the studies with different time since vaccination, respectively.

**Figure S10: Results of the data-driven multiparametric meta-regression analysis model (analysis 4)**


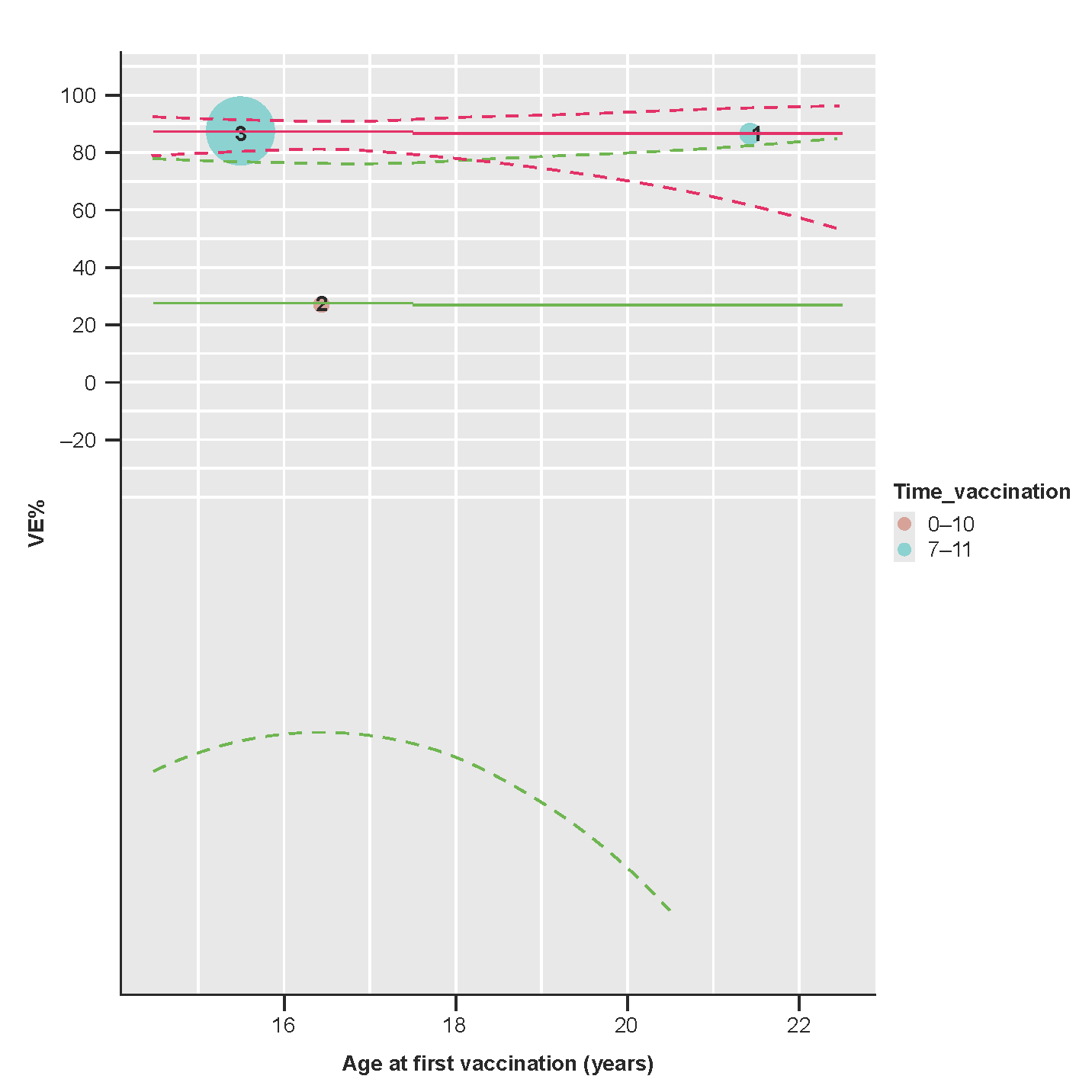


TVC=total vaccinated cohort. VE=vaccine effect.

Note for interpretation of graphs:

Subgroup:

1=Shing 2022,^2^ age at first vaccination 18–25 years, TVC, time since vaccination 7–11 years.

2=Lehtinen 2017,^6^ age at first vaccination 16–17 years, TVC, time since vaccination 0–10 years.

3=Rebolj 2022,^9^ age at first vaccination 14–17 years, TVC, time since vaccination 7–11 years.

Size of the bubbles is proportional to the inverse of the variance (corresponds to the weight in classical meta-analysis). The numbers on the bubbles represent each subgroup, with details about the age at first vaccination or the age range at the time of vaccination provided in the figure footnote.

**Analysis 5: What is the efficacy of HPV16/18-AS04 vaccine (Cervarix^®^) on CIN3+ caused by any HPV type? (RCTs only)**

**Multiparametric meta-regression**

All possible combinations of predictors were evaluated and compared using AIC (data-driven approach) to find the best model, and which predictors were the most important ones. This data-driven exploration conducted to a final model that includes the “age at first vaccination” and “analytical cohort” variables. After adjusting for the “analytical cohort”, VE decreased with age at first vaccination.

The heterogeneity explained by the selected model was R^2^=100%. This optimistic value is because the estimated between-trial variability is equal to “0”. Therefore, the interpretation of this result should be prudent. However, as shown in **Figure S11**: Results of the data-driven multiparametric meta-regression analysis model (analysis 5), the model is predicting the data very well.

The following figure shows the predictions (with 95% CIs, dotted line) from this data-driven selected model adjusting for “age at first vaccination” and “analytical cohort”. Red and green curves represent the predicted vaccine efficacy as a function of age for the time since vaccination. Red and green bubbles represent the observed vaccine efficacy of the studies with TVC and TVC-naïve population, respectively. Observed values seem to be relatively well approximated by the multiparametric model.

**Figure S11: Results of the data-driven multiparametric meta-regression analysis model (analysis 5)**

##
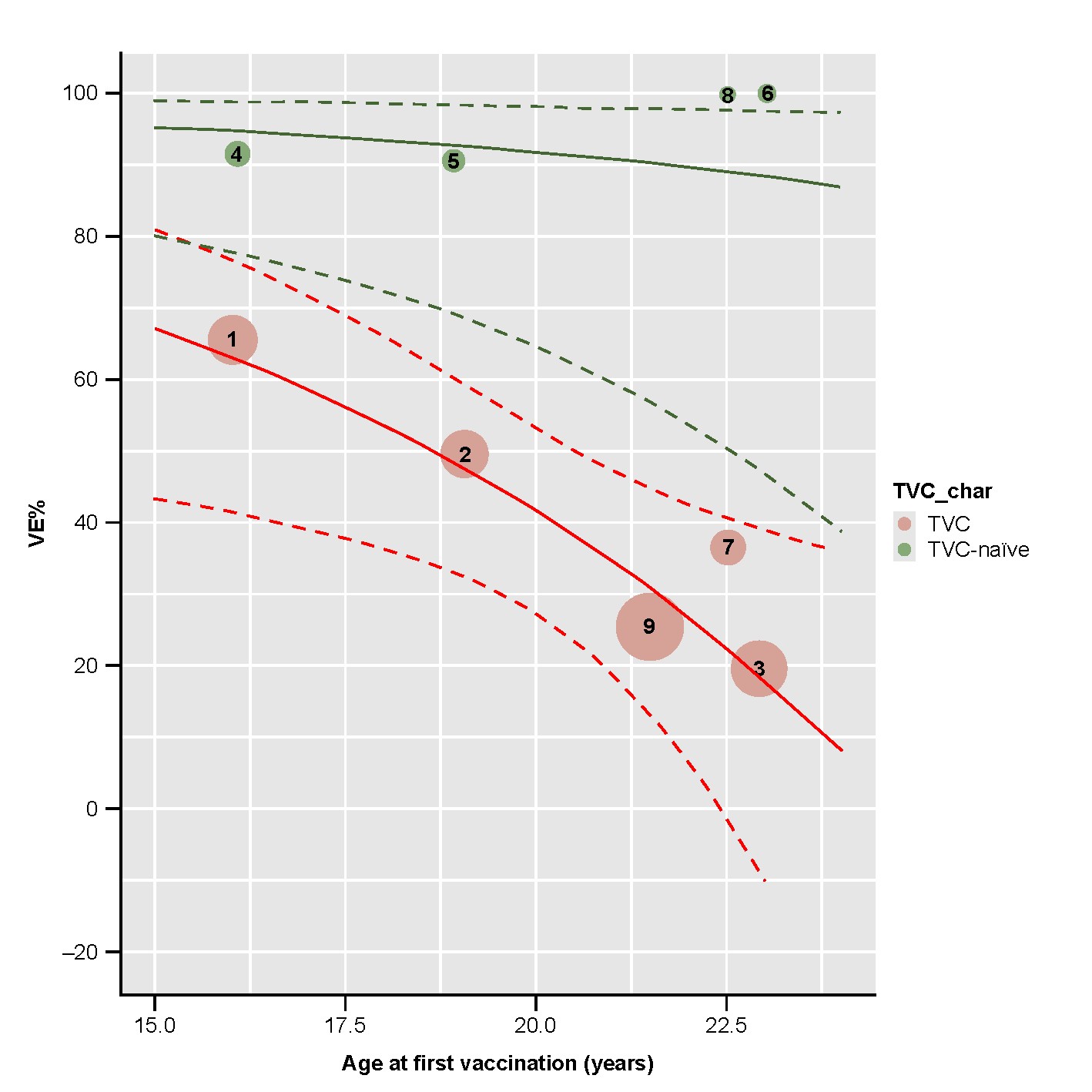


Char=characteristic. TVC=total vaccinated cohort. VE=vaccine effect.

Note for interpretation of graphs:

Subgroup

1=Lehtinen 2012,^4^ age at first vaccination 15–17 years, TVC, time since vaccination 0–4 years.

2=Lehtinen 2012,^4^ age at first vaccination 18–20 years, TVC, time since vaccination 0–4 years.

3=Lehtinen 2012,^4^ age at first vaccination 21–25 years, TVC, time since vaccination 0–4 years.

4=Lehtinen 2012,^4^ age at first vaccination 15–17 years, TVC-naïve, time since vaccination 0–4 years.

5=Lehtinen 2012,^4^ age at first vaccination 18–20 years, TVC-naïve, time since vaccination 0–4 years.

6=Lehtinen 2012,^4^ age at first vaccination 21–25 years, TVC-naïve, time since vaccination 0–4 years.

7=Konno 2014,^5^ age at first vaccination 20–25 years, TVC, time since vaccination 0–4 years.

8=Konno 2014,^5^ age at first vaccination 20–25 years, TVC-naïve, time since vaccination 0–4 years.

9=Shing 2022,^2^ age at first vaccination 18–25 years, TVC, time since vaccination 1–4 years.

Size of the bubbles is proportional to the inverse of the variance (corresponds to the weight in classical meta-analysis). The numbers on the bubbles represent each subgroup, with details about the age at first vaccination or the age range at the time of vaccination provided in the figure footnote.

**Analysis 6. What is the effectiveness of HPV16/18-AS04 vaccine (Cervarix^®^) on CIN3+ caused by any HPV type? (Observational studies only)**

**Multiparametric meta-regression analysis**

All possible combinations of predictors were evaluated and compared using AIC (data-driven approach) to find the best model, and which predictors were the most important ones. This data-driven exploration conducted to a final model that includes the “age at first vaccination” and “time since vaccination” (time of follow-up) variables. The model fitted the data well and did not present extremely high correlation between variables. After adjusting for the “time since vaccination”, vaccine effectiveness decreased with age at first vaccination. The heterogeneity explained by the selected model was R^2^=82.59%.

The following figure shows the predictions (with 95% CIs, dotted line) from this data-driven selected model adjusting for “age at first vaccination” and “time since vaccination” for two selected values of time. Red and green curves represent the predicted vaccine effectiveness as a function of age for the time since vaccination (the “0–2” years of “time since vaccination” corresponds to the red curve, and the “7–11” years of “time since vaccination” is depicted by the green curve). Colours of the different bubbles represent the observed vaccine effectiveness of the studies with different time since vaccination. As observed in the graph vaccine effectiveness decreases with age at first vaccination and it is lower among the “7–11” years of follow-up group. Even if the time of follow-up is shorter in this age group, the “0–2” years of time since vaccination group [Palmer, 2019], represents those vaccinated at older age (≥18 years) whereas the “7–11” years of follow-up group were vaccinated at a younger age (14–17 years).

**Figure S12: Results of the data-driven multiparametric meta-regression analysis model (analysis 6)**


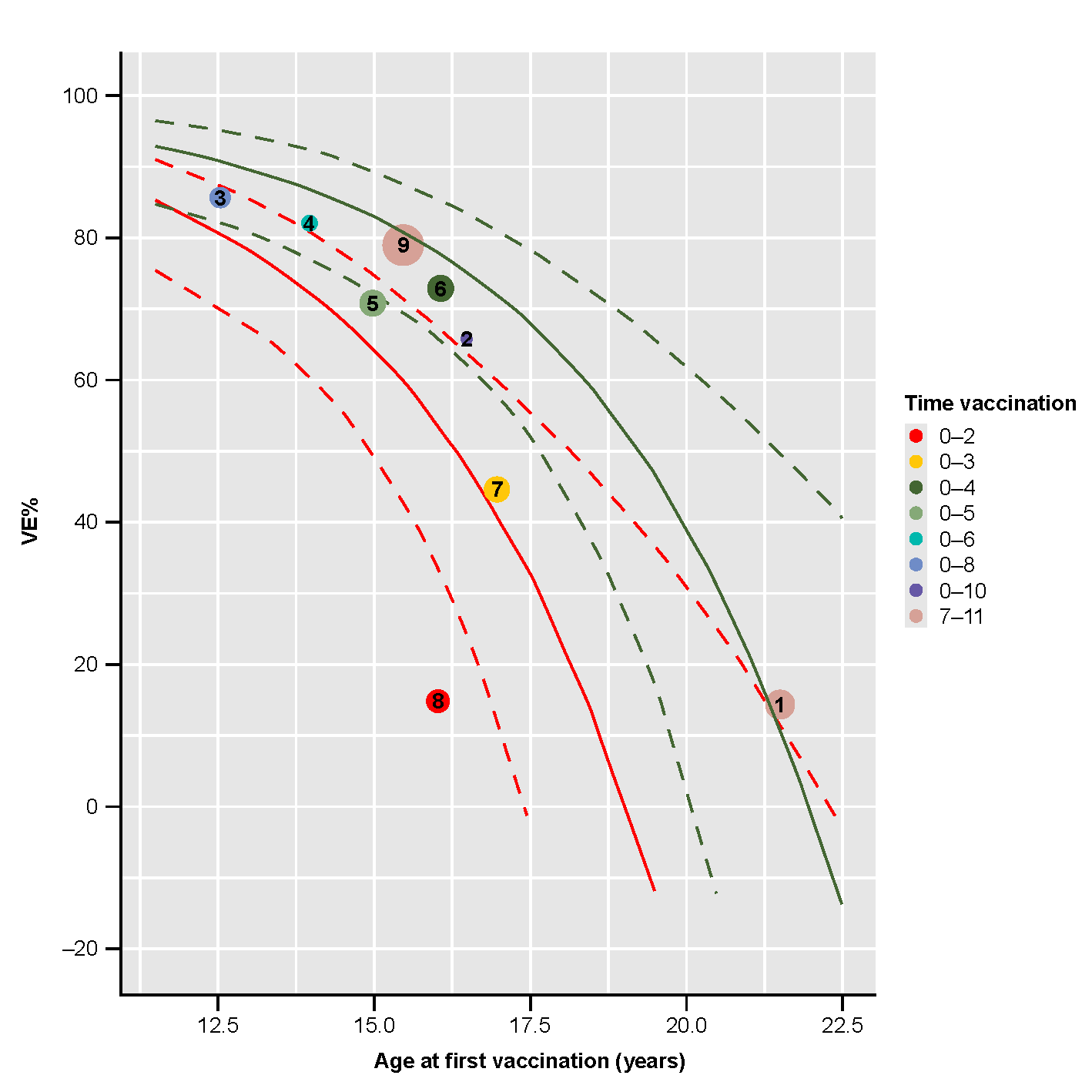


TVC=total vaccinated cohort. VE=vaccine effect.

Note for interpretation of graphs:

Subgroup

1=Shing 2022,^2^ age at first vaccination 18–25 years, TVC, time since vaccination 7–11 years.

2=Lehtinen 2017,^6^ age at first vaccination 16–17 years, TVC, time since vaccination 0–10 years.

3=Palmer 2019,^7^ age at first vaccination 12–13 years, TVC, time since vaccination 0–8 years.

4=Palmer 2019,^7^ age at first vaccination 14 years, TVC, time since vaccination 0–6 years.

5=Palmer 2019,^7^ age at first vaccination 15 years, TVC, time since vaccination 0–5 years.

6=Palmer 2019,^7^ age at first vaccination 16 years, TVC, time since vaccination 0–4 years.

7=Palmer 2019,^7^ age at first vaccination 17 years, TVC, time since vaccination 0–3 years.

8=Palmer 2019,^7^ age at first vaccination ≥18 years, TVC, time since vaccination 0–2 years.

9=Rebolj 2022,^9^ age at first vaccination 14–17 years, TVC, time since vaccination 7–11 years.

Size of the bubbles is proportional to the inverse of the variance (corresponds to the weight in classical meta-analysis). The numbers on the bubbles represent each subgroup, with details about the age at first vaccination or the age range at the time of vaccination provided in the figure footnote.

**Secondary outcomes**

Falcaro and colleagues^8^ determined the effects of HPV16/18-AS04 after its implementation as part of the NIP in England from 2008 to 2012. Results from this nationwide population-based study revealed a vaccine effectiveness on CIN3 of 97% (95% CI 96–98) and at 87% (95% CI 72–94) on cervical cancer, among the 12–13-year-old vaccinated cohort (**Table SII**).

Lehtinen and colleagues^4^ reported vaccine efficacy against HPV16/18-related AIS of 100% (95% CI 15.5–100) in the TVC-naïve, whereas it was 70% (95% CI –16.6–94.7) in the TVC. Vaccine efficacy against AIS, irrespective of HPV DNA in the lesion in the TVC-naïve and TVC, was 100% (95% CI 31.0–100) and 76.9% (95% CI 16.0–95.8), respectively (**Table SII**).

Wheeler et al investigated the vaccine efficacy on CIN3+ caused by non-vaccine types as part of the PATRICIA trial^3^ (**Table SII**). The vaccine efficacy in the TVC-naïve was 81.9% (95% CI 17.1–98.1) and 40.0% (95% CI 1.1–64.2) in the TVC, on a CIN3+ composite index of 12 HR-HPV types (31, 33, 35, 39, 45, 51, 52, 56, 58, 59, 66, and 68).

Palmer et al^7^ (**Table SII**) investigated the impact of HPV16/18-AS04 introduction in the NIP in Scotland among unvaccinated cohorts born in 1995 and 1996 (the same age than vaccine-eligible cohorts, 12–13 years old), and found a vaccine effectiveness against CIN3 estimated at 100% (95% CI 69–100) compared with unvaccinated women born in 1988–1990, confirming herd effects of the vaccine.

**References**

1. Porras C, Tsang SH, Herrero R. *et al.* Efficacy of the bivalent HPV vaccine against HPV 16/18-associated precancer: long-term follow-up results from the Costa Rica Vaccine Trial. *Lancet Oncol* 2020;**21**:1643–52.
2. Shing JZ, Hu S, Herrero R, Hildesheim A. *et al.* Precancerous cervical lesions caused by non-vaccine-preventable HPV types after vaccination with the bivalent AS04-adjuvanted HPV vaccine: an analysis of the long-term follow-up study from the randomised Costa Rica HPV Vaccine Trial. *Lancet Oncol* 2022;**23**:940–49.
3. Wheeler CM, Castellsagué X, Garland SM. *et al.* Cross-protective efficacy of HPV-16/18 AS04-adjuvanted vaccine against cervical infection and precancer caused by non-vaccine oncogenic HPV types: 4-year end-of-study analysis of the randomised, double-blind PATRICIA trial. Lancet Oncol 2012;**13**:100–10.
4. Lehtinen M, Paavonen J, Wheeler CM. *et al.* Overall efficacy of HPV-16/18 AS04-adjuvanted vaccine against grade 3 or greater cervical intraepithelial neoplasia: 4-year end-of-study analysis of the randomised, double-blind PATRICIA trial. *Lancet Oncol* 2012;13:89–99.
5. Konno R, Yoshikawa H, Okutani M. *et al*. Efficacy of the human papillomavirus (HPV)-16/18 AS04-adjuvanted vaccine against cervical intraepithelial neoplasia and cervical infection in young Japanese women. *Hum Vaccin Immunother* 2014;**10**:1781–94.
6. Lehtinen M, Lagheden C, Luostarinen T. *et al*. Ten-year follow-up of human papillomavirus vaccine efficacy against the most stringent cervical neoplasia end-point - registry-based follow-up of three cohorts from randomized trials. *BMJ Open* 2017;**7**:e015867.
7. Palmer T, Wallace L, Pollock KG. *et al.* Prevalence of cervical disease at age 20 after immunisation with bivalent HPV vaccine at age 12-13 in Scotland: retrospective population study. *BMJ* 2019;**365**:l1161.
8. Falcaro M, Castanon A, Ndlela B. *et al.* The effects of the national HPV vaccination programme in England, UK, on cervical cancer and grade 3 cervical intraepithelial neoplasia incidence: a register-based observational study. *Lancet* 2021;**398**:2084–92.
9. Rebolj M, Pesola F, Mathews C, Mesher D, Soldan K, Kitchener H. The impact of catch-up bivalent human papillomavirus vaccination on cervical screening outcomes: an observational study from the English HPV primary screening pilot. *Br J* *Cancer* 2022;127:278–87.
10. McGuinness LA, Higgins JPT. Risk-of-bias VISualization (robvis): An R package and Shiny web app for visualizing risk-of-bias assessments. *Res Synth Methods* 2021;**12**:55–61.
